# Supplementary material for: Mapping of sequences in the 5’ region and 3’ UTR of tomato ringspot virus RNA2 that facilitate cap-independent translation of reporter transcripts in vitro
Source: PLoS One. 2021 Apr 9;16(4):e0249928. doi: 10.1371/journal.pone.0249928 (PMC8034749; doi:10.1371/journal.pone.0249928)
Supplement: S1 Table — (DOCX) [file pone.0249928.s001.docx]

**S1 Table: Sequences and oligonucleotides used in the study**

| **Name** | **Sequence (5’ -3’)** | **Remarks** |
| --- | --- | --- |
| DV53_sm_F | CTAGGTCCGATGGGGATCTG | AGG^2^ mutation |
| DV53_sm_R | CCCGACGGAAAGCAGCT |  |
| DV51_df_F | ATGGCTACAAGTAAGGTCTATG | V76RV |
| DV51_df_R | CAAAAGAAAAGAAAACAAAACGGC |  |
| DV_T7V30-F1 | TAATACGACTCACTATAGGGGTTGGCTTCCTGAGAGGCG | V3' |
| DV_T7V30-R1 | GCTTTTATGAACTTGGACAAAGTTCG | V3' |
| DC_T7C_F1 | TAATACGACTCACTATAGGGCAGGGTTATTGTCTCATGAGCG | C |
| DC_T7C_R2 | CCTTGACGGCCTTCCTTCAATTC | C |
| DV31_df_F | CATTCTGGCGAGATACGCAATG | VRVΔ1 |
| DV31_df_R | GATATCCTATTGCTCGTTCTTAAGG | VRVΔ1 |
| DV32_df_F | GGTAGGACGCCATTGTTC | VRVΔ2 |
| DV32_df_R | GGACTCACCCACATAAGATTTATC | VRVΔ2 |
| DV33_df_F | TTAACTTTAGCTGTAATGTAGTGG | VRVΔ3, VRVΔ3d |
| DV33_df_R | TCTGGTACTAAGTTCGTTTCAG | VRVΔ3, VRVΔ3a |
| DV34_df_F | GTCCAAGTTCATAAAAGCTCTAGAAAAAAA | VRVΔ4 |
| DV34_df_R | AATTCTAGATCGTACCGCATGCTGG | VRVΔ4 |
| DV_o33_F1 | TAGGATATCGGTAGGACGCCATTGTTCCAGG | VRVΔ124 |
| DV_o33_R1 | AACCTCTAGATCGTACCGCATGCTGGCCTT | VRVΔ124 |
| DV33-1-F1 | GCTCCCGGTTCTTTCTTAC | VRVΔ3a |
| DV33-2-R1 | CCCTGGATAGTACACGAATA | VRVΔ3b |
| DV33-2-F1 | TTTCTACACGTTAGTGTTATGACG | VRVΔ3b |
| DV33-3-F1 | GTACTGTTGACGGAGGAGTA | VRVΔ3c |
| DV33-3-R1 | ACACGACATATTCCTTCAGTG | VRVΔ3c |
| DV33-4-R1 | ACATGCAATTGTGTCGTACG | VRVΔ3d |
