# Supplementary material for: Mapping of sequences in the 5’ region and 3’ UTR of tomato ringspot virus RNA2 that facilitate cap-independent translation of reporter transcripts in vitro
Source: PLoS One. 2021 Apr 9;16(4):e0249928. doi: 10.1371/journal.pone.0249928 (PMC8034749; doi:10.1371/journal.pone.0249928)
Supplement: S2 Table — The nucleotide sequence surrounding in-frame AUG codons is shown (positions -3 to +5). Positions that correspond to the plant consensus start codon context: (G/A)(A/C)aAUGGC are underlined. C or U at the -3 position and/or A, C or U at the +4 position are rare in this consensus context and are highlighted in yellow. Predicted stem-loops downstream from AUGs are listed if they are less than 23 nts away from the AUG and have a free energy ΔG equal to or lower than -10. The distance to the AUG (in nts) and the calculated free energy (ΔG) of the stem-loops are indicated in the parentheses. Only the first AUG is listed if it is placed in an optimal conserved context or if a stem-loop with acceptable free energy is predicted at a location 10 to 23 nts downstream of the AUG. When the first AUG does not meet these conditions, subsequent AUGs within the first 1200 nts of the RNA are listed (up to the first four), until they meet these conditions. The NCBI accession number is shown for each sequence. Abbreviations are as follows: ToRSV (tomato ringspot virus), BRV (blackcurrant reversion virus), CLRV (cherry leaf roll virus), PRMV (peach rosette mosaic virus), BLSV (blueberry latent spherical virus), SLSV (soybean latent spherical virus), GBLV (grapevine Bulgarian latent virus), ArMV (arabis mosaic virus), GFLV (grapevine fanleaf virus), RRSV (raspberry ringspot virus), TRSV (tobacco ringspot virus), MMLRaV (mulberry mosaic roll leaf-associated virus), GDefV (grapevine deformation virus), PBRSV (potato black ringspot virus), MMMoV (melon mild mottle virus), TBRV (tomato black ring virus), CNSV (cycas necrotic stunt virus), AILV (artichoke Italian latent virus). (DOCX) [file pone.0249928.s002.docx]

**S2 Table. Putative translation initiation sites in selected nepovirus RNAs**

The nucleotide sequence surrounding in-frame AUG codons is shown (positions -3 to +5). Positions that correspond to the plant consensus start codon context: (G/A)(A/C)aAUGGC are underlined. C or U at the -3 position and/or A, C or U at the +4 position are rare in this consensus context and are highlighted in yellow. Predicted stem-loops downstream from AUGs are listed if they are less than 23 nts away from the AUG and have a free energy **ΔG** equal to or lower than -10. The distance to the AUG (in nts) and the calculated free energy (ΔG) of the stem-loops are indicated in the parentheses. Only the first AUG is listed if it is placed in an optimal conserved context or if a stem-loop with acceptable free energy is predicted at a location 10 to 23 nts downstream of the AUG. When the first AUG does not meet these conditions, subsequent AUGs within the first 1200 nts of the RNA are listed (up to the first four), until they meet these conditions. The NCBI accession number is shown for each sequence. Abbreviations are as follows: ToRSV (tomato ringspot virus), BRV (blackcurrant reversion virus), CLRV (cherry leaf roll virus), PRMV (peach rosette mosaic virus), BLSV (blueberry latent spherical virus), SLSV (soybean latent spherical virus), GBLV (grapevine Bulgarian latent virus), ArMV (arabis mosaic virus), GFLV (grapevine fanleaf virus), RRSV (raspberry ringspot virus), TRSV (tobacco ringspot virus), MMLRaV (mulberry mosaic roll leaf-associated virus), GDefV (grapevine deformation virus), PBRSV (potato black ringspot virus), MMMoV (melon mild mottle virus), TBRV (tomato black ring virus), CNSV (cycas necrotic stunt virus), AILV (artichoke Italian latent virus)

**SUBGROUP C NEPOVIRUSES**

ToRSV-Rasp1 RNA1 (KM083894) GCAAUGUU (17 nts, ΔG -18)

ToRSV-Rasp1 RNA2 (KM083895) UUGAUGUC (17 nts, ΔG -19)

BRV RNA1 (NC_003509) ACAAUGGU (10 nts, ΔG -10)

BRV RNA2 (NC_003502) ACCAUGAG (15 nts, ΔG -14)

CLRV RNA1 (NC_015414) AAAAUGGU

CLRV RNA2 (NC_015415) AAAAUGGU

PRMV RNA1 (NC_034214) CUUAUGGA (16 nts, ΔG -11)

PRMV RNA2 (NC_034215) AAAAUGCU (20 nts, ΔG -12)

BLSV RNA1 (NC_038764) AUUAUGGA

BLSV RNA2 (NC_038763) UCCAUGAU; augaugac; cggaugau (20 nts, ΔG -15)

SLSV RNA1 (NC_032270) GCAAUGUC; gauaugcg (4 nts, ΔG -14); gcuaugug; aagauggc

SLSV RNA2 (NC_032271) AACAUGGU; gccaugaa (19 nts, ΔG -19)

GBLV RNA1 (NC_015492) AACAUGGC

GBLV RNA2 (NC_015493) UUGAUGUG (13 nts, ΔG -15)

**SUBGROUP A NEPOVIRUSES**

ArMV RNA1 (NC_006057) ACUAUGUG (17 nts, ΔG -18)

ArMV RNA2 (NC_006056) CUUAUGGC (16 nts, ΔG -16)

GFLV RNA1 (NC_003615) ACUAUGUG (12 nts, ΔG -26)

GFLV RNA2 (NC_003623) CUUAUGGG (17 nts, ΔG -12)

RRSV RNA1 (NC_005266) ACAAUGGG

RRSV RNA2 (NC_005267) ACAAUGUC (3 nts, ΔG -18); GUCAUGGA (17 nts, ΔG -20)

TRSV RNA1 (NC_005097) AAGAUGGG (20 nts, ΔG -22)

TRSV RNA2 (NC_005096) CCGAUGGA; GCAAUGAC; UUCAUGAC (-2 nts, ΔG -11); GAAAUGAC

MMLRaV RNA1 (NC_038767) GUUAUGGG (15 nts, ΔG -13)

MMLRaV RNA2 (NC_038768) CGUAUGGA; UUUAUGGG (11 nts, ΔG -14)

GDefV RNA1 (NC_017939) ACUAUGUG (18 nts, ΔG -15)

GDefV RNA2 (NC_017938) CGUAUGGG; UGUAUGGA; GCCAUGCU (10 nts, ΔG -14)

PBRSV RNA1 (NC_022798) UUAAUGGG (20 nts, ΔG -17)

PBRSV RNA2 (NC_022799) CCAAUGGA; GCUAUGGC

MMMoV RNA1 (NC_038765) UGCAUGCG; AAUAUGGC

MMMoV RNA2 (NC_03766) UUGAUGGC; CGUAUGGG (-1 nts, ΔG -10); CGUAUGCC

**SUBGROUP B NEPOVIRUSES**

TBRV RNA1 (NC_004439) GACAUGUC; CCGAUGUC; CCCAUGGA; UACAUGGG

TBRV RNA2 (NC_004440) CUAAUGUC; ACAAUGGG

CNSV RNA1 (NC_003791) UUGAUGUC (22 nts, ΔG -12)

CNSV RNA2 (NC_003792) GAAAUGUU; GGCAUGGG

AILV RNA1 (NC_043684) CUUAUGCG; CCCAUGGA; GAAAUGAU (18 nts, ΔG -12)

AILV RNA2 (NC_043685) AUUAUGGG
