# Supplementary material for: Mapping of sequences in the 5’ region and 3’ UTR of tomato ringspot virus RNA2 that facilitate cap-independent translation of reporter transcripts in vitro
Source: PLoS One. 2021 Apr 9;16(4):e0249928. doi: 10.1371/journal.pone.0249928 (PMC8034749; doi:10.1371/journal.pone.0249928)
Supplement: S1 Fig — The sequences of ToRSV RNAs were aligned using Clustal Omega and alignment results are depicted for the ~900 initial nucleotides. The three putative start codons are shown in red. Sequences at the 5’ end of the RNA complementary to predicted loops in region 3a-b are highlighted in light blue (see Figs 9 and S4 for more details). The predicted complementary stem sequences of a putative stem-loop (5’ SL) structure located after the first AUG are shown with arrows. Labelling of ToRSV RNAs and NCBI accession numbers are as in Fig 2. (DOCX) [file pone.0249928.s003.docx]

S1 Fig. **Sequence alignment of the 5’ region of the genomic RNAs of selected ToRSV isolates.** The sequences of ToRSV RNAs were aligned using Clustal Omega and alignment results are depicted for the ~900 initial nucleotides. The three putative start codons are shown in red. Sequences at the 5’ end of the RNA complementary to predicted loops in region 3a-b are highlighted in light blue (see **Fig 9** and **S4 Fig** for more details). The predicted complementary stem sequences of a putative stem-loop (5’ SL) structure located after the first AUG are shown with arrows. Labelling of ToRSV RNAs and NCBI accession numbers are as in **Fig 2**.

GYV RNA1 UUGCGAAACUUGGAUACUUAUUCCAAAUUCUCUCUUUUCUUUUCUUAUCACAAACAAUUU 60

GYV RNA2 UUGCGAAACUUGGAUACUUAUUCCAAAUUCUCUCUUUUCUUUUCUUAUCACAAACAAUUU 60

13C280 RNA1 UUGCGAAAAAUCUGGUGAUAUUCCAAGUUCUCUCAAUUCACACUUCCAU----------- 49

13C280 RNA2 UUGCGAAAAAGCUGGUGAUAUUCCAAGUUCUCUCAAUUCACACUUCCA-----------U 49

Rasp1 RNA1 UUGCGAAACUUUCGGUGAUAUUCCGAAUUCUCUUUUCUCACUUUUAAUU----------- 49

Rasp1 RNA2 UUGCGAAAAAUCUGGUGAUAUUCCAAGUUCUCUCGAUUUACACUUCCA-----------U 49

********. . ******.*.****** * * .

GYV RNA1 CCUUGUGUUUUGAAUCGUUUUGUUUUGAUGUUUUC---AAGUUUUUGUGAUGGCAGUCAC 117

GYV RNA2 CCUUGUGUUUUGAAUUGUUUUGUUUUGAUGUUCUC---AAGUUUUUGUGAUGGCAGUCAC 117

13C280 RNA1 UGUGCCGUUUUGUUUUCUUUUCUUCUGAUGUCCUCCAUUUGUUUCGUUGGUGGCAACCAU 109

13C280 RNA2 UGUGCCGUUUUGUUUUCUUUUCUUCUAAUGUCUUCCAUCUGUUUCGCUGGUGGCAACCAU 109

Rasp1 RNA1 UGUGCUGCUUUGAUUUCUUUUCGCA--AUGUUUUCAGAUUGUCAUGGUGGUGGCAACCAU 107

Rasp1 RNA2 UGUGCCGUUUUGUUUUCUUUUCUUUUGAUGUCCUCCAUUUGUUUCGCCGGUGGCAACCAC 109

* * **** * **** **** ** ** *.*****. **

AUG^1^

GYV RNA1 GCUAGACUGCCAUCAAAGGCUGCUUUCCGUCGGCUUAUCGCCGAUGGAGAUUUGGAUCGU 177

GYV RNA2 GCUAGACUGCCAUCAAAGGCUGCUUUCCGUCGGCUUAUUGCCGAUGGAGAUUUGGAUCGU 177

13C280 RNA1 GCUAGGUUGCCAUCAAAAGCUGCUUUCCGUCGGGCUAUGUCCGAUGGGGAUCUGGACCGC 169

13C280 RNA2 GCUAGGUUGCCAUCGAAAGCUGCUUUCCGUCGGGCUAUGUCCGAUGGGGAUCUGGACCGC 169

Rasp1 RNA1 GCUAGGUUGCCAUCAAAGGCUGCUUUCCGUCGGGCUAUGUCCGACGGAGAUCUUGAUCGC 167

Rasp1 RNA2 GCUAGGUUGCCAUCGAAAGCUGCUUUCCGUCGGGCUAUGUCCGAUGGGGAUCUGGACCGC 169

*****. *******.**.*************** *** **** **.*** * ** **

5’SL AUG^2^

GYV RNA1 GAGGGACGCUACCCUUGCGGUUGCCUUGCACAAUAUUUUGUGCAAUCUGCUCCUGCCCCG 237

GYV RNA2 GAGGGACGCUACCCUUGCGGUUGCCUUGCACAAUAUUUUGUGCAAUCUGCUCCUGCCCCG 237

13C280 RNA1 GAGGGUCGCUUCCCUUGCGGGUGUCUAGCACAGUAUGCUGUGCAAGUCCCCCCUCCUGCC 229

13C280 RNA2 GAGGGUCGCUUCCCUUGCGGGUGUCUAGCACAGUAUGCUGUGCAAGUCCCCCCUCCUGCC 229

Rasp1 RNA1 GAGGGUCGCUUCCCUUGCGGGUGUCUUGCACAGUUUGAUGUGCAAAUCCCCUCUCCUGCA 227

Rasp1 RNA2 GAGGGUCGCUUCCCUUGCGGGUGUCUAGCACAGUAUACUGUGCAAGCCCCCCCUCCUGCC 229

***** **** ********* ** ** *****.* * ******* * ** * *

GYV RNA1 AAGGCACAGGAGGCGGUUGCCCCCCGUAAAGUGGGUGUCGUAGGUAGGUCCGCUGACCUC 297

GYV RNA2 AAGGCACAGGAGGCGGUUGCCCCCCGUAAAGUGGGUGUCGUAGGUAGGUCCGCUGACCUC 297

13C280 RNA1 AAGACACAGGAGACA---------------------GUCGUAGGCAGGUCCGCUGACCUC 268

13C280 RNA2 AAGACACAGGAGACA---------------------GUCGUAGGCAGGUCCGCUGACCUC 268

Rasp1 RNA1 AAGGCACAGGAGAAA---------------------GUCGUAGGUAGGUCCGCUGACCUC 266

Rasp1 RNA2 AAGACACAGGAGACA---------------------GUCGUAGGCAGGUCCGCUGACCUC 268

***.********... ******** ***************

GYV RNA1 CA---GGGUGCUGUUGCUCCCCUUAAGAAGCAACGCUGUGAUGUUGUGGUCGCAGUUGCU 354

GYV RNA2 CA---GGGUGCUGUUGCUCCCCUUAAGAAGCAACGCUGUGAUGUUGUGGUCGCAGUUUCU 354

13C280 RNA1 CGAAAGGGUAAUGUUGCUCCCCUUAAGAAGCAACGCUGCGAUGUUGUGGUCGCAGUUUCU 328

13C280 RNA2 CAAAAGGGUAAUGUUGCUCCCCUUAAGAAGCAACGCUGCGAUGUUGUGGUCGCAGUUUCU 328

Rasp1 RNA1 CA---GGGUAAUGUUGGUCCCCUCAAGAAGCAACGCUGCGAUGUUGUGGUCGCAGUUUUU 323

Rasp1 RNA2 CAAAAGGGUAAUGUUGCUCCCCUUAAGAAGCAACGCUGCGAUGUUGUGGUCGCAGUCUCU 328

*. ****..***** ****** ************** ***************** *

GYV RNA1 GGACCUCCUCCACUGGAGUUGGUCUACCCUGCCCGGGUAGGGCAACAUAGGUUGGAUCAG 414

GYV RNA2 GGACCUCCUCCACUGGAGUUGGUCUACCCUGCCCGGAUAGGGCAACAUAGGUUGGAUCAG 414

13C280 RNA1 GGACCUCCUCCUUUGGAGUUGGUCUACCCUGCCCGGGUAGGGCAACAUAGGUUGGACCAA 388

13C280 RNA2 GGACCUCCUCCUUUGGAGUUGGUCUACCCUGCCCGGGUAGGGCAACAUAGGUUGGACCAA 388

Rasp1 RNA1 GGACCUCCUCCGUUGGAGUUGGUUUACCCGGCCCGGGUAGGGCAACAUAGGUUGGACCAA 383

Rasp1 RNA2 GGACCUCCUCCUUUGGAGUUGGUCUACCCUGCCCGGGUAGGGCAGCAUAGGUUGGACCAA 388

*********** ********** ***** ******.*******.*********** **.

GYV RNA1 CCUUCUACUGGUCCCUUGGCAGUUCCUGCUGCCAAGCAAACUUCCGCUGCAACGGGAGUU 474

GYV RNA2 CCUUCUACUGGUCCCUUGGCAGUUCCUGCUGCCAAGCAAACUUCCGCUGCAACGGAAGUU 474

13C280 RNA1 ACCUCAAAAGGUCCCUUGGCAGUCCCCUCUGCCAAGCAAACCUCCACUGCAAUGGAGGUU 448

13C280 RNA2 ACCUCAAAAGGUCCCUUGGCAGUCCCCUCUGCCAAGCAAACCUCCACUGCAAUGGAGGUU 448

Rasp1 RNA1 CCUUCAAAAGGUCCCUUGGCAGUUCCUGCUGCCAAGCAAACCUCCACUGCAAUGGAGGUU 443

Rasp1 RNA2 CCUUCAAAAGGUCCCUUGGCAGUCCCCUCUGCCAAGCAAACCUCCACUGCAAUGGAGGUU 448

.* ** *. ************** ** ************* ***.****** **..***

AUG^3^

GYV RNA1 GUUCUUUCUGUCAGGGAGGCAGCUCUUACUGCCCCUUGGCUCCUUCGCUCCUGCAAGAGC 534

GYV RNA2 GUUCUUUCUGUCAGGGAGGCAGCUCUUACUGCCCCUUGGCUCCUUCGCUCCUGCAAGAGC 534

13C280 RNA1 GUUCUUUCUGUCCAGGAGGCGGCUAUCACUGCCCCCUGGCUUCUUCGCCCCUGCAAGGGU 508

13C280 RNA2 GUUCUUUCUGUCCAGGAGGCGGCUAUCACUGCCCCCUGGCUUCUUCGCCCCUGCAAGGGU 508

Rasp1 RNA1 GUUCUUUCUGUUAGGGAGGCAGCUUUCACCGCCCCCUGGCUCCUUCGCUCCUGCAAGAGC 503

Rasp1 RNA2 GUUCUUUCUGUCCAGGAGGCGGCUAUCACUGCCCCCUGGCUUCUUCGCCCCUGCAAGGGU 508

*********** ..******.*** * ** ***** ***** ****** ********.*

GYV RNA1 GAAGUUUCUUCUAUCCCCCCCCCUCUUUCCCAAAGGCAGCAAUUUGCUGCCAUUAAAAGG 594

GYV RNA2 GAAGUUUCUUCUAUCCCCCCCCCUCUUUCUCAAAGGCAGCAAUUUGCUGCCAUUAAAAGA 594

13C280 RNA1 GAAGCUUCCCCCCC---CCCCCCCCUUACACAAAGGCAGCAAUUCGCUGCCCUAAAGAAG 565

13C280 RNA2 GAAGCUUCCCC---CCCCCCCCCCCUUACACAAAGGCAGCAAUUCGCUGCCCUAAAGAAG 565

Rasp1 RNA1 GGAGUUCCCCCCCCCCCCCCCCCCAUGACACAAAGGCAGCAGUUUGCUGCCUUGAAGAGG 563

Rasp1 RNA2 GAAGCUUCCCC---CCCCCCCCCCCUUACACAAAGGCAGCAAUUCGCUGCCCUAAAGAAG 565

*.** * * * ****** .* * ***********.** ****** * **.*..

GYV RNA1 AGGCUGACCCUAAAAGGUCAGCAAAUCAUUCGCGAGCACAUUCGUGCUCGCAAAGCGGCA 654

GYV RNA2 AGGCUGACCCUAAAAGGUCAGCAAAUUAUUCGCGAGCACAUUCGUGCUCGCAAAGCGGCA 654

13C280 RNA1 AGGCUGGCCGCCAAGGGCCAGCAAAUCAUCCGCGAGCACAUUCGUGCUCGCAAGGCGGCU 625

13C280 RNA2 AGGCUGGCCGCCAAGGGCCAGCAAAUCAUCCGCGAGCACAUUCGUGCUCGCAAGGCGGCU 625

Rasp1 RNA1 AGGCUGGUCCAAAAGGGCCAGCAAACUAUUCGCGAGCUCAUCCGAGCUCGCAAGGCGGCU 623

Rasp1 RNA2 AGGCUGGCCGCCAAGGGCCAGCAAAUUAUCCGCGAGCACAUUCGUGCUCGCAAGGCGGCC 625

******. * .**.** ******* ** ******* *** ** ********.*****

GYV RNA1 AAGUAUGCCGCUUUUGCUGCAGCCAAGAGGGCUGCGGCUCUUGCUGCCCAAAAGGCGGCA 714

GYV RNA2 AAGUAUGCCGCUUUUGCUGCAGCCAAGAGGGCUGCAGCUCUUGCUGCCCAAAAGGCGGCA 714

13C280 RNA1 AAAUACGCCGCCAUUGCCAAAGCCAAGAAGGCUGCGGCUUCUGCUGCCGUUAAGGCAGCG 685

13C280 RNA2 AAAUACGCCGCCAUUGCCAAAGCCAAGAAGGCUGCGGCUUCUGCUGCCGUUAAGGCAGCG 685

Rasp1 RNA1 AAGUAUGCCGCCAUUGCCGCCCAGAAAAGGGCGGCUGCUGUGGCUGCCCAAAAGGCAGCA 683

Rasp1 RNA2 AAAUAUGCCGCCAUCGCCAAAGCCAAAAAGGCUGCGGCUCUUGCUGCCGUUAAGGCAGCG 685

**.** ***** * ** ... . **.*.*** ** *** ****** *****.**.

GYV RNA1 GCAGAGGCUUCACGCCUCGCGGCCCAAAGGGCCGCAGUUGCCAAAUUCCUCCGGGAUAGG 774

GYV RNA2 GCAGAGGCUUCACGCCUCGCGGCCCAAAGGGCCGCAGUUGCCAAAUUCCUCCGGGAUAGG 774

13C280 RNA1 CAGGAGGCUCCCCGCCUCGCGGCCCAAAAGGCCGCCAUCAGCAAGAUCCUUAGGGAUCGA 745

13C280 RNA2 CAGGAGGCUCCCCGCCUCGCGGCCCAAAAGGCCGCCAUCAGCAAGAUCCUUAGGGAUCGA 745

Rasp1 RNA1 GCUGGGGCCCCGCGCCUCGCGGCCCAAAGGGCCGCAGUUGCUAAGAUCCUUCGGGAUCGG 743

Rasp1 RNA2 CAGGAGGCUCCUCGCCUUGCGGCCCAAAAGGCUGCCAUCAGCAAGAUCCUUAGGGAUCGA 745

. *.*** * ***** **********.*** **..* . **. **** .*****.*.

GYV RNA1 CAACUUGCCUCUUUUCCCCCUCCCCCUUCCCCAUCUGCUGCCCAAUUGGCAGCCGAGGCU 834

GYV RNA2 CAACUUGCCUCUUUUCCCCCUCCCCCUCCCCCUUCCGCUGCCCAAUUGGCAGCCGAGGCU 834

13C280 RNA1 GCUGUUGCUGCUCUCCCCCCUCCCCCUACUCCUUCUGCUGCCAGAUUGGCAGCUGAGGCC 805

13C280 RNA2 GCUGUUGCUGCUCUCCCCCCUCCCCCUACUCCUUCUGCUGCCAGAUUGGCAGCUGAGGCC 805

Rasp1 RNA1 CAACUGGCUUCUCUUCCCCCUCCUCCUCCUCCUUCUGCUGCCAGAUUGGCAGCUGAGGCC 803

Rasp1 RNA2 GCUGUUGCUGCUCUCCCCCUUCCUCCUCCUCCUUCUGCUGCCAGAUUGGCAGCUGAGGCC 805

. * ** ** * **** *** *** * ** ** ******..********* *****

GYV RNA1 GAAUUGGCCUCGAAAGCUGAGUCUCUUCGGAGGCUCAAGGCCUUUCGAAAGGCCAGCAGG 894

GYV RNA2 GAAUUGGCCUCGAAAGCUGAGUCUCUUCGGAGGCUCAAGGCCUUUCAAAAGGCCAGCAGG 894

13C280 RNA1 GAAUUGGCCUCAAAGGCCGAGUCUCUUCGGAGGCUCAAGGCCUUUCACAAGUCCAGCAGG 865

13C280 RNA2 GAAUUGGCCUCAAAGGCCGAGUCUCUUCGGAGGCUCAAGGCCUUUCACAAGUUCAGCAGG 865

Rasp1 RNA1 GAAUUGGCCUCUAAAGCAGCCUCUCUUUUGAGGCUAAAGGCCUUUAAAAAGGCCAGCAGG 863

Rasp1 RNA2 GAAUUGGCCUCAAAGGCCGAGUCUCUUCGGAGGCUCAAGGCCUUUCGCAAGUUCAGCAGG 865

*********** **.** *. ****** ******.*********...*** *******

GYV RNA1 GUUCGCCCUGCUCUGAAUAAUUCUAUUCCUUCUCCCCCUCU---CAAAAGGCAGCGGGAA 951

GYV RNA2 GUUCGCCCUGCUUUAAAUAAUUCCUUUCCUUCCCCUCC---UCCUCCUGUAGUUCGCUCU 951

13C280 RNA1 GUUCGCCCUGCUUUAAACAACUCUUUUCCCUCCCCCCCUUU---GGCGCGCAAGCCAGAU 922

13C280 RNA2 GUUCGCCCUGCUUUAAAUACUUCUUUUCUUUCUCUUCCCCCUCCCCCUCCAGCUCGGUCC 925

Rasp1 RNA1 GUUCGCCCUGCUUUAAAUAUUUUUUUUCCUCUCCCCCCCCC---UGAGCGGGUUCGCGAU 920

Rasp1 RNA2 GUUCGCCCUGCUUUAAACACUUCUUUUCUUUCUCCUCCCCCUUCCCCUCCGGCUCGGUCU 925

************ *.** * * *** * ** . * .

GYV RNA1 GCUGCCCUUCUCGAAAGGUUGCGUAUUG--------------CAACCCCCUCUCGCUCCU 997

GYV RNA2 UGCGAACUGCUGGCAGCCUUUAAGGCUGCCAUGAACAGGUCUCAGCCUGUUCAGGGGGGU 1011

13C280 RNA1 UCCGCUCUUCUCGAGCGGUUGAGGCUUG--------------CUACGCCUUCACGCUCUA 968

13C280 RNA2 UCCGAGCUUUUGGCAGCUUUUAGUGCUGCCAUGAACAGGUCUCAGCCUGUUCAAGGGGGU 985

Rasp1 RNA1 CCCGCCCUUCUUGAGCGUCUGAGGGUGG--------------CAACACCCUCACGCGCCC 966

Rasp1 RNA2 UCCGAGCUUUUGGCGGCUUUUAGUGCCGCCAUGAACAGGUCUCAGCCUGUUCAAGGGGGU 985

*. ** * *.. * .. * * .* ** *
