# Supplementary material for: Mapping of sequences in the 5’ region and 3’ UTR of tomato ringspot virus RNA2 that facilitate cap-independent translation of reporter transcripts in vitro
Source: PLoS One. 2021 Apr 9;16(4):e0249928. doi: 10.1371/journal.pone.0249928 (PMC8034749; doi:10.1371/journal.pone.0249928)
Supplement: S2 Fig — The sequences of ToRSV RNAs 3’ UTRs were aligned using Clustal Omega. Stop codons are indicated in red. The start point of each deletion mutant is indicated with small black arrows: R1 (region 1), R2 (region 2), R3/R3a (region 3 and region 3a), R3b (region 3b), R3c (region 3c), R3d (region 3d) and R4 (region 4). Sequences complementary to the 5’ end of the RNA are highlighted in light blue (see Figs 9 and S4 for more details). (DOCX) [file pone.0249928.s004.docx]

S2 **Fig. Sequence alignment of the 3’ UTRs of the genomic RNAs of selected ToRSV isolates.** The sequences of ToRSV RNAs 3’ UTRs were aligned using Clustal Omega. Stop codons are indicated in red. The start point of each deletion mutant is indicated with small black arrows: R1 (region 1), R2 (region 2), R3/R3a (region 3 and region 3a), R3b (region 3b), R3c (region 3c), R3d (region 3d) and R4 (region 4). Sequences complementary to the 5’ end of the RNA are highlighted in light blue (see Fig 9 and S4 Fig for more details).

GYV RNA1 ----**UAG**UUUAGCUCUUUAAGAGCGGAGUGGCUGCCCUAAGCAGCCUCCAAAAGGUGGCC 6743

GYV RNA2 **UGA**UUAGUUUAGCUCUUUAAGAGCGGAGUGGCUGCCCUAAGCAGCCUCCAAAAGGUGGCC 5805

13C280 RNA1 ----**UAA**UUC----CUCUUAGAGGCGAGUAGCUGCCGUUAGCAGCUUCCAAAAGGUGGCC 6716

13C280 RNA2 ----**UAA**GUUGGCUUCCUGAAAGGCGAGUAGCUGCCGUUAGCAGCUUCCAAAAGGUGGCC 5778

Rasp1 RNA1 ----**UGA**GGUUUUUUUAGGUUAGGCGAGUAGCUGCCGUAAGCAGCUUCCAAUAGGUGGCC 6712

Rasp1 RNA2 ----**UAA**GUUGGCUUCCUGAGAGGCGAGUAGCUGCCGUUAGCAGCUUCCAAAAGGUGGCC 6069

* ** **** ****** * ****** ***** ********

R1

GYV RNA1 UCUUAAUUAGCUUUU-AAUAGGGGUUAUCCAGCCUUGAGCAAGCUGGCACCGGUGCUGAU 6802

GYV RNA2 UCUUAAUUAGCUUUU-AAUAGGGGUUAUCCAGCCUUGAGCAAGCUGGCACCGGUGCUGAU 5864

13C280 RNA1 UCUUAUUUAGCUUUU-AAUAGGGGUUAUCCAGCCUUAAGCAAGCUGGCACCGGUCCUGAU 6775

13C280 RNA2 UCUUAUUUAGCUUUU-AAUAGGGGUUAUCCAGCCUUAAGCAAGCUGGCACCGGUCCUGAU 5837

Rasp1 RNA1 UCUUAAUUAGCUUUGAAAUAGGGGUUAUCUAGCCUUGAGCAAGCUGGCACCGGUCCUGAU 6772

Rasp1 RNA2 UCUUAAUUAGCUUUU-AAUAGGGGUUAUCCAGCCUUAAGCAAGCUGGCACCGGUCCUGAU 6128

***** ******** ************* ****** ***************** *****

GYV RNA1 GCACUACCAGGGAAGUACCUGGUU-UGGAAGAAUUC-AGAUAAGAUUCUUAAAUCUUGCU 6860

GYV RNA2 GCACUACCAGGGAAGUACCUGGUU-UGGAAGAAUUC-AGAUAAGAUUCUUAAAUCUUGCU 5922

13C280 RNA1 GGACUACCAGGAAAGUACCUGGUU-UGGAAGAAUUC-GAGUAAAAUUCUUAAAUCUUGUU 6833

13C280 RNA2 GGACUACCAGGAAAGUACCUGGUU-UGGAAGAAUUC-GAGUAAAAUUCUUAAAUCUUGUU 5895

Rasp1 RNA1 GGACUACCAGGAAAGUACCUGGUUUUGGAAGAAUUUGUAGUGGGAUUCUUAAAUCUUGCC 6832

Rasp1 RNA2 GGACUACCAGGAAAGUACCUGGUU-UGGAAGAAUUC-GAGUAAAAUUCUUAAAUCUUGUU 6186

* ********* ************ ********** * **************

GYV RNA1 UAUCUGU---GACUAAUGACAAGUUCAAAAAGAACUGGUCCAUGUUUGCU--GCUUGCAU 6915

GYV RNA2 UAUCUGU---GACUAAUGACAAGUUCAAAAAGAACUGGUCCAUGUUUGCU--GCUUGCAU 5977

13C280 RNA1 UGCUCGU---GACUUAUAGUACAUUCAAGAGGAAUGACUC-AUGUUUUGUCCAUUUACAU 6889

13C280 RNA2 UGCUCGU---GACUUAUAGUACAUUCAAGAGGAAUGACUC-AUGUUUUGUCCAUUUACAU 5951

Rasp1 RNA1 UGCUACUAGGAGCCUUUAGUAAGCUCUG--------AAUC--UAUUUACUCCCAAAAGGU 6882

Rasp1 RNA2 UACUCGU---GACUUAUAGUACAUUCAAGAUGAAUGACUC-AUGUUUUGUCCAUUUACAU 6242

* * * * * ** ** * *** * *

GYV RNA1 GAUGGCAGGGAGGUAU-----------GAUGGCA--CGCGUAUUGCUCCUUACGUUCAAG 6962

GYV RNA2 GAUGGCAGGGAGGUAU-----------GAUGGCA--CGCGUAUUGCUCCUUACGUUCAAG 6024

13C280 RNA1 GAUGGCAUAAAGAGUUAACGGCU-------------CAUAUGGCGCUCAUUACGUUCAAG 6936

13C280 RNA2 GAUGGCAUAAAGAGUUAACGGCU-------------CAUAUGGCGCUCAUUACGUUCAAG 5998

Rasp1 RNA1 GAAGC-GAAACUAGCCAUCUCCUUGGAAAAGGUGUGUGUAAGUGCUUAAUUUCCUUCGGG 6941

Rasp1 RNA2 GAUGGCAUAAAGAGUUAACGGCU-------------CAUAUGGCGCUCAUUACGUUCAAG 6289

** * * ** * *** *

GYV RNA1 UGUUGAAGGGUUUAAUAGCCUUGAACUGUGGU------AAUACUUGUGGUGACCUGCAUC 7016

GYV RNA2 UGUUGAAGGGUUUAAUAGCCUUGAACUGUGGU------AAUACUUGUGGUGACCUGCAUC 6078

13C280 RNA1 UGUUGAAGGAUCCAAUAGCCUUGAACUGUGG----UGCCAUGUGAGGAGAUCC--ACGUU 6990

13C280 RNA2 UGUUGAAGGAUCCAAUAGCCUUGAACUGUGG----UGUCAUGUGAGGAGAUCC--ACGUU 6052

Rasp1 RNA1 CCGUUGUGGAUCCUAUAACGGAGUU-UGUAGAAUAUAUGACACUAGGAAAGACUAGCGUU 7000

Rasp1 RNA2 UGUUGAAGGAUUCAAUAGCCUUGAACUGUGG----UGCCAUGUGAGGAAAUCC--ACGUU 6343

* ** * *** * * *** * * * * * *

GYV RNA1 A--UCCCAAUCUGUCAAAGCAGAUAGUCUAGGAGACG-----AUAAAUCCUAUGUGGGUA 7069

GYV RNA2 A--UCCCAAUCUGUCAAAGCAGAUAGUCUAGGAGACG-----AUAAAUCCUAUGUGGGUA 6131

13C280 RNA1 AUC-UCUGAUUGUCAAAAUAGACUAGUCUAGGAGACG-----AUAAAUCCUAUGUGGGUG 7044

13C280 RNA2 AUC-UCUGAUUGUCAAAAUAGACUAGUCUAGGAGACG-----AUAAAUCCUAUGUGGGUG 6106

Rasp1 RNA1 UUAUAGUGAUUGACGUAAAUAAUCA-CUAAGGACACCUGGAGAGACAGGCGCUUUGGCA- 7058

Rasp1 RNA2 AUC-UCUGAUUGUCAAAAUAGACUAGUCUAGGAGACG-----AUAAAUCUUAUGUGGGUG 6397

** ** * **** ** * * * * ***

GYV RNA1 AGUCCCACACUGACG--AGUCACGUGAAAACCUUUUAUUCAUUUGUGGUUAACAAAUGUC 7127

GYV RNA2 AGUCCCACACUGACG--AGUCACGUGAAAACCUUUUAUUCAUUUGUGGUUAACAAAUGUC 6189

13C280 RNA1 AGUCCCAUUCUGGCG--AGACAC-GCAAUGCCUUUUAUUUGUUUGAGGUUAUCAAACAUC 7101

13C280 RNA2 AGUCCCAUUCUGGCG--AGACAC-GCAAUGCCUUUUAUUUGUUUGAGGUUAUCAAACAUC 6163

Rasp1 RNA1 --GC----UCAUGCGUUAUCGAC-GCUGCUACCUUUAUUUGUUUGGGG-AAACAAACAUC 7110

Rasp1 RNA2 AGUCCCAUUCUGGCG--AGAUAC-GCAAUGCCUUUUAUUUGUUUGAGGUUAUCAAACAUC 6454

* * ** * ** * ****** **** ** * **** **

R2

GYV RNA1 AUAUUUUGAGUCUGCAUUUUAUUUU-UAAUAAUGUAGAUCGAGUUUAGUCUACCGAUGAG 7186

GYV RNA2 AUAUUUUGAGUCUGCAUUUUAUUUU-UAAUAAUGUAGAUCGAGUUUAGUCUACCGAUGAG 6248

13C280 RNA1 AUAUUUUGAGUCUGCAUUUAAAUUC-CAAUAAUGUAGUUGUC--AUAGCCUACCGAUGAA 7158

13C280 RNA2 AUAUUUUGAGUCUGCAUUUAAAUUC-CAAUAAUGUAGUUGUC--AUAGCCUACCGAUGAA 6220

Rasp1 RNA1 AUAUCUUGAGUCUGCAUUCAAAUUUAUAAUAAUGUAGUUGUC--AUAGCCUACCGAUGAG 7168

Rasp1 RNA2 AUAUCUUGAGUCUGCGUUUAAAUUU-CAAUAAUGUAGUUGUC--AUAGCCUACCGAUGAA 6511

**** ********** ** * ** ********** * *** **********

GYV RNA1 GCUGCGAGAACGGUUCCCUUAAGGCAUAUUCUCAGAAAGGGAUUAAGUUGCUGUCUAUGU 7246

GYV RNA2 GCUGCGAGAACGGUUCCCUUAAGGCAUAUUCUCAGAAAGGGAUUAAGUUGCUGUCUAUGU 6308

13C280 RNA1 CCUGCGAGAAAGGUUCCAUGAGGACUAGGG------------UUGGCUAACCCU------ 7200

13C280 RNA2 CCUGCGAGAAAGGUUCCAUGAGGACUAGGG------------UUGGCUAACCCU------ 6262

Rasp1 RNA1 CCUGCGAGAAAGGUUCCAUGAAGACUAGAG------------UUGGCUAACUCC------ 7210

Rasp1 RNA2 CCUGCGAGAAAGGUUCCAUGAGGACUAGGG------------UUGGCUAACCCU------ 6553

********* ****** * * * * ** * *

GYV RNA1 GUCAUGAUUUUUCUGAUGAGAGUACUUUCAGAAUUAUGUGAUUGACAUCUUGGCUAAGGU 7306

GYV RNA2 GUCAUGAUUUUUCUGAUGAGAGUACUUUCAGAAUUAUGUGAUUGACAUCUUGGCUAAGGU 6368

13C280 RNA1 CACUUAAUCUCUCUAUUGGUC----AUUCGACAGUGCGU---CGAGAAUUUA---UGGGU 7250

13C280 RNA2 CACUUAAUCUCUCUAUUGGUC----AUUCGACAGUGCGU---CGAGAAUUUA---UGGGU 6312

Rasp1 RNA1 CACUUAAUCUCUCUAUUGAUC----AUUCGACAGUGUGU---UGAGAACUUA---UGGGU 7260

Rasp1 RNA2 CACUUAAUCUCUCUAUUGGUC----AUUCGACAGUGUGU---CGAGAAUUCA---UGGGU 6604

* * ** * *** ** *** * * ** ** * * ***

GYV RNA1 UUUCUUAAAGAUCUUCAUGGUUAUUCAGUUAAGGAUAUUCUGCUUGGGUGUCCCAGCAGG 7366

GYV RNA2 UUUCUUAAAGAUCUUCAUGGUUAUUCAGUUAAGGAUAUUCUGCUUGGGUGUCCCAGCAGG 6428

13C280 RNA1 UUCAUCACCCACAUUGAAG----------CGAGUGUCUCGU-----AAGAAACCACUCGG 7295

13C280 RNA2 UUCAUCACCCACAUUGAAG----------CGAGUGUCUCGU-----AAGAAACCACUCGG 6357

Rasp1 RNA1 UUUAUCACCUAUGGUGGAG----------CGGUUGCAACCCAACUGUAGAUGUCACUUGG 7310

Rasp1 RNA2 UUCAUCACCCACAUUGAAG----------CGAGUGUCUCGU-----AAGAAACCACUCGG 6648

** * * * * * ** **

GYV RNA1 AUGCAAAGGAUUGCCUCCCAGUAGCGCAUGUCUGUAGCCCUCAAGCGAACGCCAUACCCG 7426

GYV RNA2 AUGCAAAGGAUUGCCUCCCAGUAGCGCAUGUCUGUAGCCCUCAAGCGAACGCCAUACCCG 6488

13C280 RNA1 AUUGAUGUACUUACCAUGCAUCCUUUCGAGUA---AAGCAUCGAUUCGUCGUUGUGGUUC 7352

13C280 RNA2 AUUGAUGUACUUACCAUGCAUCCUUUCGAGUA---AAGCAUCGAUUCGUCGUUGUGGUUC 6414

Rasp1 RNA1 AUUGAGGCACUCACCACGCUUCAUUUCAAGUA---AAGUAUCGAUUCGUCGGUAUGAUUC 7367

Rasp1 RNA2 AUUGGUGUACUUACCAUGCAUCCUUUCGAGUA---AAGCAUCGAUUCGUCGUUGUGGUUC 6705

** * ** * * ** * ** * ** *

GYV RNA1 AUCAAACAUAUUUCUCAAGCGAACGCCAUACCCGGAAGGAUUGGCCAUGUAACUUGUAAG 7486

GYV RNA2 AUCAAACAUAUUUCUCAAGCGAACGCCAUACCCGGAAGGAUUGGCCAUGUAACUUGUAAG 6548

13C280 RNA1 UUCAAUUGUGGUUUUAG-AUGAGCGAUGAGUU------CGCUGGCCGCGUU-------AG 7398

13C280 RNA2 UUCAAUUGUGGUUUUAG-AUGAGCGAUGAGUU------CGCUGGCCGCGUU-------AG 6460

Rasp1 RNA1 UCCA-UUAUAGCUCUUG-GUAAACGGUAAGUU------CAUUGAUCGCGUU-------AG 7412

Rasp1 RNA2 UUCAAUUGUGGUUUUAG-AUGAGCGAUGAGAU------CGCUGGCCGCGUU-------AG 6751

** * * * * ** ** * ** **

GYV RNA1 UGUGUCCCUGUGAGGAUUGGUUAUGUAACUUUGAGUGAGCGUCCGAUGCGUGAAGUGAGU 7546

GYV RNA2 UGUGUCCCUGUGAGGAUUGGUUAUGUAACUUUGAGUGCGCGUCCGAUGCGUGAAGUGAGU 6608

13C280 RNA1 ------AGCGUGAAAAGUAGUC-UGAAA---------CGAACUUAGUAUCAGAGGUAGGA 7442

13C280 RNA2 ------AGCGUGAAAAGUAGUC-UGAAA---------CGAACUUAGUAUCAGAGGUAGGA 6505

Rasp1 RNA1 ------AGUGUGGAAAAUAGUC-UGAAA---------CGAACUCAGUACCAGAGGUAGGA 7457

Rasp1 RNA2 ------AGCGUGAAAAGUAGUC-UGAAA---------CGAACUUAGUACCAGAGGUAGGA 6795

*** * * ** ** ** * * ** ** *

R3/R3a

GYV RNA1 GCAUGUUUGCAUCAUUUCUAUAAUUGAAUUUUCCAUUUAGGGGAAAUUUAGACAGCAACC 7606

GYV RNA2 GCAUGUUUGCAUCAUUUCUAUAAUUGAAUUUUCCAUUUAGGGGAAAUUUAGACAGCAACC 6668

13C280 RNA1 CGCC---------AUUGUUCCAG--GCGUU-UUUUUUUUUGG---GCAUAAGCUGUAAAU 7487

13C280 RNA2 CGCC---------AUUGUUCCAG--GCGUU-UUU--UUUUGG---GCAUAAGCUGUAAAU 6547

Rasp1 RNA1 CGCU---------AUUGUUCCAG--GCGUU-UC---UUAUGG---GCAUAAGCUGUAAAC 7498

Rasp1 RNA2 CGCC---------AUUGUUCCAG--GCGUU-UU---UUAUGG---GCAUAAGCUGUAAAU 6837

*** * * * ** * ** ** ** * * **

GYV RNA1 UCUAUACCCUAGGGUUCCAUGCUUAUAACGAACUUAGUAUCAGAGGUAGAACGCUAUUGU 7666

GYV RNA2 UCUAUACCCUAGGGUUCCAUGCUUAUAACGAACUUAGUAUCAGAGGUAGAACGCUAUUGU 6728

13C280 RNA1 UUGGUUUCGCAAGCCAUUCAGCACCUCCCU-UAUUC-------GUGUACUAUCCAGGGGC 7539

13C280 RNA2 UUGGUUUCGCAAGCCAUUCAGCACCUCCCU-UAUUC-------GUGUACUAUCCAGGGGC 6599

Rasp1 RNA1 UUGGUUUCGCAAGCCAUUCAGCACCUCCCUUUGCUU-------GUGUACUAUCUAGGGGC 7551

Rasp1 RNA2 UUGGUUUCGCAAGCCAUUCAGCACCUCCCUUUAUUC-------GUGUACUAUCCAGGGGC 6890

* * * * * ** * * * *** * *

R3b

GYV RNA1 UCCAGGCG-UCUCUUAUGGACAUAAGCUGUAGAUGGGGCUUCGCAAACCCUGCAGCUCUU 7725

GYV RNA2 UCCAGGCG-UCUCUUAUGGACAUAAGCUGUAGAUGGGGCUUCGCAAACCCUGCAGCUCUU 6787

13C280 RNA1 UCCCGGUUCUUUCUUACCGGUACAAUACCUG-----------GCGAAGCGAAU------- 7581

13C280 RNA2 UCCCGGUUCUUUCUUACCGGUACAAUACCUG-----------GCGAAGCGAAU------- 6641

Rasp1 RNA1 UCCCGGCC-UUU-CUUCCGGUACAAUACCUA-----------GUGAAGCAAGCA------ 7592

Rasp1 RNA2 UCCCGGUUCUUUCUUACCGGUACAAUACCUG-----------GCGAAGCGAAU------- 6932

*** ** * * * * * ** * * ** *

GYV RNA1 CCGAUUAGCGGAAGGAAAAGAGUAGCAUGUUCCUGCUUAGUGAAGGAAUAUGUCGUGUUU 7785

GYV RNA2 CCGAUUAGCGGAAGGAAAAGAGUAGCAUGUUCCUGCUUAGUGAAGGAAUAUGUCGUGUUU 6848

13C280 RNA1 --AUUGCGUCGAGGGAUGAGAGUAGCAUGUUCCUGCUCAUUGAAGGAAUAUGUCGUGUUU 7639

13C280 RNA2 --AUUGCGUCGAGGGAUGAGAGUAGCAUGUUCCUGCUCAUUGAAGGAAUAUGUCGUGUUU 6699

Rasp1 RNA1 --AUUGCGUUGAGGGAUAAGAGUAGCAUGUUCCUGCUUAACGGAGGAAUAUGUCGUGUUU 7650

Rasp1 RNA2 --AUUGCGUCGAGGGAUGAGAGUAGCAUGUUCCUGCUCACUGAAGGAAUAUGUCGUGUUU 6990

* * ** *** ******************* * * *****************

R3c

GYV RNA1 GCCACACGUUAGUGUUGCAAUGCUGUAAUGGCAUUGUAGUGCAGGUAUGGUUCCCAGCCA 7845

GYV RNA2 GCCACACGUUAGUGUUGCAAUGCUGUAAUGGCAUUGUAGUGCAGGUAUGGUUCCCAGCCA 6907

13C280 RNA1 UCUGCACGUUAGUGUUAUGACGCUACCC-AGCGCCAUAGUGCAAGAAUGGUUCCCAGCCA 7698

13C280 RNA2 UCUGCACGUUAGUGUUAUGACGCUAUCC-AGCGCCAUAGUGCAAGAAUGGUUCCCAGCCA 6758

Rasp1 RNA1 UCUACACGUUAGUGUUGCAUUGCUAUAAUGGCAAUGUAGUGCAGGAAUGGUUCCCAGCCA 7710

Rasp1 RNA2 UCUACACGUUAGUGUUAUGACGCUACCC-AGCGCCAUAGUGCAAGAAUGGUUCCCAGCCA 7049

* ************ *** ** ******* * **************

GYV RNA1 CUUU-UUCUGGGAUUCUAAUCGUACGACACAAAUUGCAUGUGUAUUGCUGACGGAGGAGU 7904

GYV RNA2 CUUU-UUCUGGGAUUCUAAUCGUACGACACAAAUUGCAUGUGUAUUGCUGACGGAGGAGU 6966

13C280 RNA1 CUUU-UUCUGGGAUUCUAAUCGUACGACACA-AUUGCAUGUGUACUGUUGACGGAGGAGU 7756

13C280 RNA2 CUUU-UUCUGGGAUUCUAAUCGUACGACACA-AUUGCAUGUGUACUGUUGACGGAGGAGU 6816

Rasp1 RNA1 CUUUUUUCUGGGAUUCUAAUCGUACGUCACA-AUUGUGUGUGUAUCGUUGACGGAGGAGU 7769

Rasp1 RNA2 CUUU-UUCUGGGAUUCUAAUCGUACGACACA-AUUGCAUGUGUACUGUUGACGGAGGAGU 7107

**** ********************* **** **** ****** * ************

R3d

GYV RNA1 AGCGACCCUCUACCACGCAAGUCCGGAAGUGAUUACCGGGGCCGAAGAAGGCCAGCAUGC 7964

GYV RNA2 AGCGACCCUCUACCACGCAAGUCCGGAAGUGAUUACCGGGGCCGAAGAAGGCCAGCAUGC 7026

13C280 RNA1 AGCGAUCCUCUAUCACGCAGGGCCGGAAGUAAUUCCCGGGGCCGAAGAAGGCCAGCAUGC 7816

13C280 RNA2 AGCGAUCCUCUAUCACGCAGGGCCGGAAGUAAUUCCCGGGGCCGAAGAAGGCCAGCAUGC 6876

Rasp1 RNA1 AGCGAUCCUCUACCACGCGAGUCUGGAAGUGAUUACCAGGGCCUAAGAUGGCCAGCACAC 7829

Rasp1 RNA2 AGCGAUCCUCUAUCACGCAGGGCCGGAAGUAAUUCCCGGGGCCGAAGAAGGCCAGCAUGC 7167

***** ****** ***** * * ****** *** ** ***** **** ******** *

GYV RNA1 GGUACGAUUAAAUUUAGCUGUAAUGUAGUGGUAUGUUAAGUUGAGACUAACUUACCCGUA 8024

GYV RNA2 GGUACGAUUAAAUUUAGCUGUAAUGUAGUGGUAUGUUAAGUUGAGACUAACUUACCCGUA 7086

13C280 RNA1 GGUACGAUUAACUUUAGCUGUAAUGUAGUGGUAUGUUAAGUUGAGACUAACUUACCCGUA 7876

13C280 RNA2 GGUACGAUUAACUUUAGCUGUAAUGUAGUGGUAUGUUAAGUUGAGACUAACUUACCCGUA 6936

Rasp1 RNA1 GGUACGAUUAAAUUUAGUUGUAAUGUAGUGGUAUGUUAAGUUGAGACUAACUUACCCGUA 7889

Rasp1 RNA2 GGUACGAUUAACUUUAGCUGUAAUGUAGUGGUAUGUUAAGUUGAGACUAACUUACCCGUA 7227

*********** ***** ******************************************

R4

GYV RNA1 CGAGUCAAACUUUGUGAUGGAUGUGUGUUCUGCCAUCAUAAGGGAAGUAGAUGUGGUUUU 8084

GYV RNA2 CGAGUCAAACUUUGUGAUGGAUGUGUGUUCUGCCAUCAUAAGGGAAGUAGAUGUGGUUUU 7146

13C280 RNA1 CGAGUCAAACUCCUUGGUGGAUGUGUGUUCUGCCACCUUGGAGGAAGUAGAUGUGAUUUU 7936

13C280 RNA2 CGAGUCAAACUCCUUGGUGGAUGUGUGUUCUGCCACCUUGGAGGAAGUAGAUAUGAUUUU 6996

Rasp1 RNA1 CGAGUCAAACUUUAAGAUGGAUGUGUGUUCUGCCAUCUUGAGGGAAGUAGAUGUGGUUUU 7949

Rasp1 RNA2 CGAGUCAAACUUUAAGAUGGAUGUGUGUUCUGCCAUCUUGAGGGAAGUAGGUGUGGUUUU 7287

*********** * ****************** * * ******** * ** ****

GYV RNA1 GCCAAUCUGAGACGAGCCAUUAAUUUGGUGCUUUAACACGUUGAUGAUAAUACUCGUGCA 8144

GYV RNA2 GCCAAUCUGAGACGAGCCAUUAAUUUGGUGCUUUAACACGUUGAUGAUAAUACUCGUGCA 7206

13C280 RNA1 ACCAGUCUGAGAUGAGCCAUUAAUUUGGUGCUUCCAUUCAUUGAUGAUAAUACUCGUGCA 7996

13C280 RNA2 ACCAGUCUGAGAUGAGCCAUUAAUUUGGUGCUUUUAUUCAUUGAUGAUAAUACUCGUGCA 7057

Rasp1 RNA1 ACCAAUCUGAGACGAGCCGUUAAUUCGGUGCUUUAAUACGUCAAUGAUAAUACUCGUGCA 8009

Rasp1 RNA2 ACCAAUCUGAGACGAGCCGUUAAUUCGGUGCUUUAAUACGUCAAUGAUAAUACUCGUGCA 7347

*** ******* ***** ****** ******* * * * *****************

GYV RNA1 GUUGCAGCUGUACGAGUAUGUUGGUACACACAGUCUACUCGGAUACGGUCGAGUUGCCCU 8204

GYV RNA2 GUUGCAGCUGUACGAGUAUGUUGGUACACACAGUCUACUCGGAUACGGUCGAGUUGCCCU 7266

13C280 RNA1 GUUGCAGCUGCACGAGUAUGUUGGUACGCAUAGUCUACUCGGAUACGGUCGAGUUGCCCU 8056

13C280 RNA2 GUUGCAGCUGCACGAGUAUGUUGGUACGCAUAGUCUACUCGGAUACGGUCGAGUUGCCCU 7116

Rasp1 RNA1 GUUGCAGCUGCACGAGUAUGUUGGUACACACAGUCUACUCGGAUACGGUCGAGUUGCCCU 8069

Rasp1 RNA2 GUUGCAGCUGCACGAGUAUGUUGGUACACACAGUCUACUCGGAUACGGUCGAGUUGCCCU 7407

********** **************** ** *****************************

GYV RNA1 CACAACGGGGAUUACUCUCUCAAUCUUAACUACUGCUAGGACGUUGUUUUCGCAGGGUUU 8264

GYV RNA2 CACAACAGGGAUUACUCUCUCAAUCUUAACUACUGCUAGGACGUUGUUUUCGCAGGGUUU 7326

13C280 RNA1 CACAACAGGGAUUA-UCUCUCAAUCUUAACUACUGCCAGGACGUUGUUUUCGCAGGGUUU 8115

13C280 RNA2 CACAACAGGGAUUA-UCUCUCAAUCUUAACUACUGCCAGGACGUUGUUUUCGCAGGGUUU 7175

Rasp1 RNA1 CACAACAGGGAUUACUCUCUCAAUCUUAACUACUGCAAGGACGUUGUUUUCGCAGGGUUU 8130

Rasp1 RNA2 CACAACAGGGAUUACUCUCUCAAUCUUAACUACUGCAAGGACGUUGUUUUCGCAGGGUUU 7467

****** ******* ********************* ***********************

GYV RNA1 UGUUGGUCCGUUUGUGUUUCAAAACGCUGCUUUGCAAUUUUCU--UUUUGUUUUAUUGCU 8322

GYV RNA2 UGUUGGUCCGUUUGUGUUUCAAAACGCUGCUUUGCAAUUUUCU--UUUUGUUUUAUUGCU 7384

13C280 RNA1 UGUUGGUCCGUUUGUGUUUCAAAACGCUGCUUUGCAUUUUUU--AUUUUGUUUUAUUGCU 8173

13C280 RNA2 UGUUGGUCCGUUUGUGUUUCAAAACGCUGCUUUGCAUUUUUC--AUUUUGUUUUAUUGCU 7233

Rasp1 RNA1 UGUUGGUCCGCUUGUGUUUCAAAACGCUGCUUUGCAAUUUUCUU-UUUUGUUUUAUUGCU 8188

Rasp1 RNA2 UGUUGGUCCGUUUGUGUUUCAAAACGCUGCUUUGCAAUUUUCUUUUUUUGUUUUAUUGCU 7527

********** ************************* **** ***************

GYV RNA1 UUCGUAGUGUCGAACCUAGUCCAGGUUUAUAAAAGC 8358

GYV RNA2 UUCGUAGUGUCGAACCUAGUCCAGGUUUAUAAAAGC 7420

13C280 RNA1 UUCGUAGUGUCGAACUUUGUCCAAGUUCAUAAAAGC 8209

13C280 RNA2 UUCGUAGUGUCGAACUUUGUCCAAGUUCAUAAAAGC 7269

Rasp1 RNA1 UUCGUAGUGUCGAACUUUGUCCAAGUUCAUAAAAGC 8224

Rasp1 RNA2 UUCGUAGUGUCGAACUUUGUCCAAGUUCAUAAAAGC 7563

*************** * ***** *** ********
