# Supplementary material for: Mapping of sequences in the 5’ region and 3’ UTR of tomato ringspot virus RNA2 that facilitate cap-independent translation of reporter transcripts in vitro
Source: PLoS One. 2021 Apr 9;16(4):e0249928. doi: 10.1371/journal.pone.0249928 (PMC8034749; doi:10.1371/journal.pone.0249928)
Supplement: S3 Fig — VRV transcripts were added to in vitro translation reactions at the concentrations of 0.05, 0.1, 0.2, 0.4, 0.8, 1, 2, 4 or 5 picomoles per 50 μl WGE and the resulting luminescence were measured. Experiments were repeated twice, each with three technical repeats, with similar results. A representative result is shown. Error bars represent the standard deviation of the three technical repeats. (PPTX) [file pone.0249928.s005.pptx]

## Slide 1
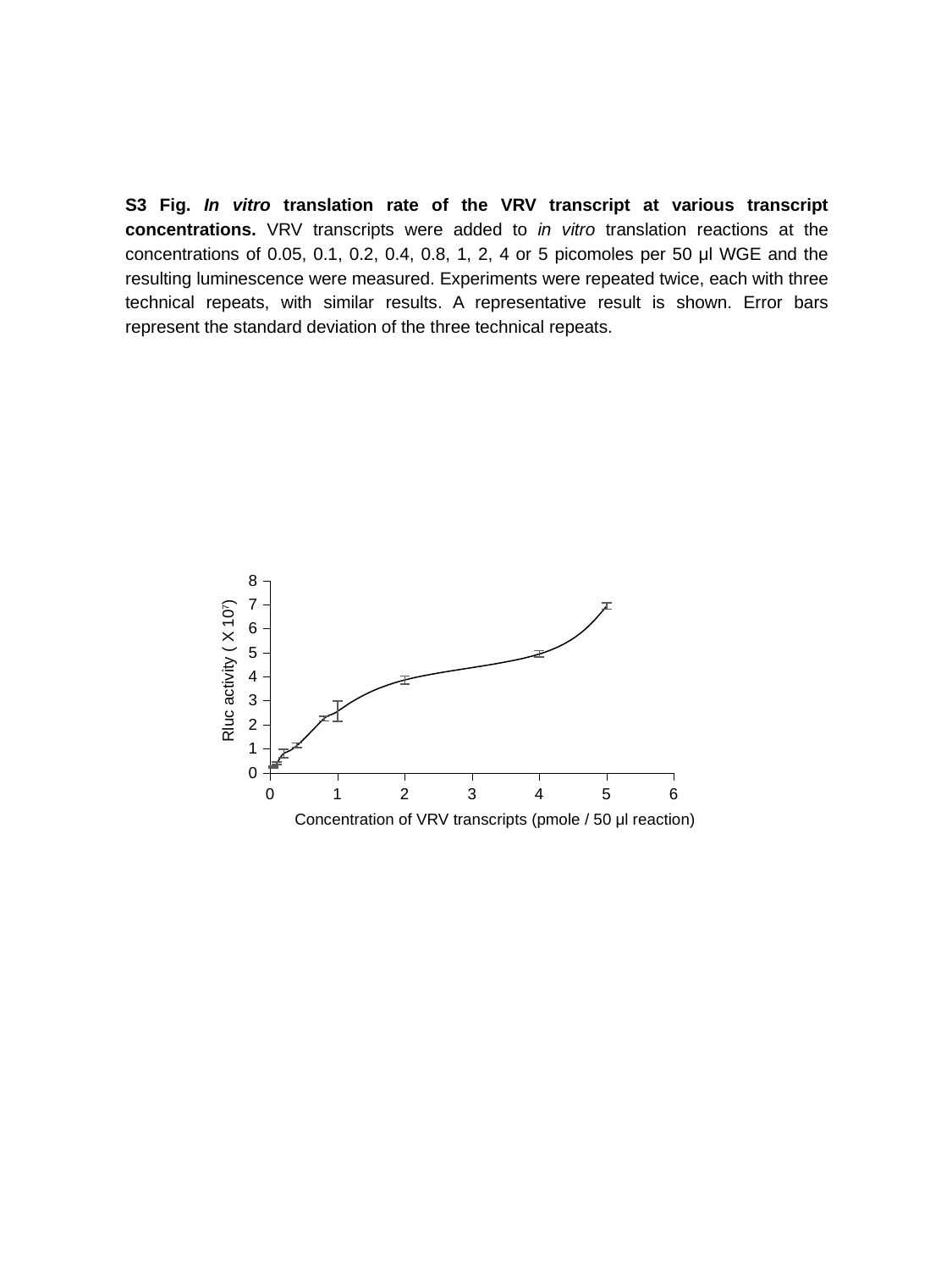

S3 Fig. In vitro translation rate of the VRV transcript at various transcript concentrations. VRV transcripts were added to in vitro translation reactions at the concentrations of 0.05, 0.1, 0.2, 0.4, 0.8, 1, 2, 4 or 5 picomoles per 50 μl WGE and the resulting luminescence were measured. Experiments were repeated twice, each with three technical repeats, with similar results. A representative result is shown. Error bars represent the standard deviation of the three technical repeats.
### Chart
| Category | |
|---|---|Rluc activity ( X 107)
Concentration of VRV transcripts (pmole / 50 μl reaction)
