# Supplementary material for: Mapping of sequences in the 5’ region and 3’ UTR of tomato ringspot virus RNA2 that facilitate cap-independent translation of reporter transcripts in vitro
Source: PLoS One. 2021 Apr 9;16(4):e0249928. doi: 10.1371/journal.pone.0249928 (PMC8034749; doi:10.1371/journal.pone.0249928)
Supplement: S4 Fig — The predicted secondary structures are shown for the RNAs of each isolate (Rasp1, 13C280, GYV) as indicated. The 5’ region spans from the first nucleotide of the RNA to the nucleotide corresponding to the position of the third AUG codon in ToRSV-Rasp1 RNA2 (see alignment in Fig 2). Region 3a-b correspond to the region aligning with the corresponding region of ToRSV-Rasp1 RNA2 (see alignment in S2 Fig). Please note that regions 3a-b of ToRSV-GYV RNA1 and RNA2 are identical. In frame AUG codons are shown in green. The position of the 5’ SL located downstream of the first AUG is shown for each RNA. Putative base pairing (sequence complementarity) between the 5’ region and region 3a-b of the 3’ UTR are indicated in light and dark blue. The light blue base pairing between a predicted loop in region 3a-b and the 5’ end of the RNA is conserved for each isolate and is also shown in Fig 9. The putative dark blue base pairing between predicted exposed bulges in two stems is only conserved in isolates Rasp1 and 13C280 and is not shown in Fig 9. Secondary structures were predicted and visualized as described in Fig 9. Polypyrimidine stretches present in exposed loops or bulges in the predicted structures are highlighted in yellow. (PPTX) [file pone.0249928.s006.pptx]

## Slide 1
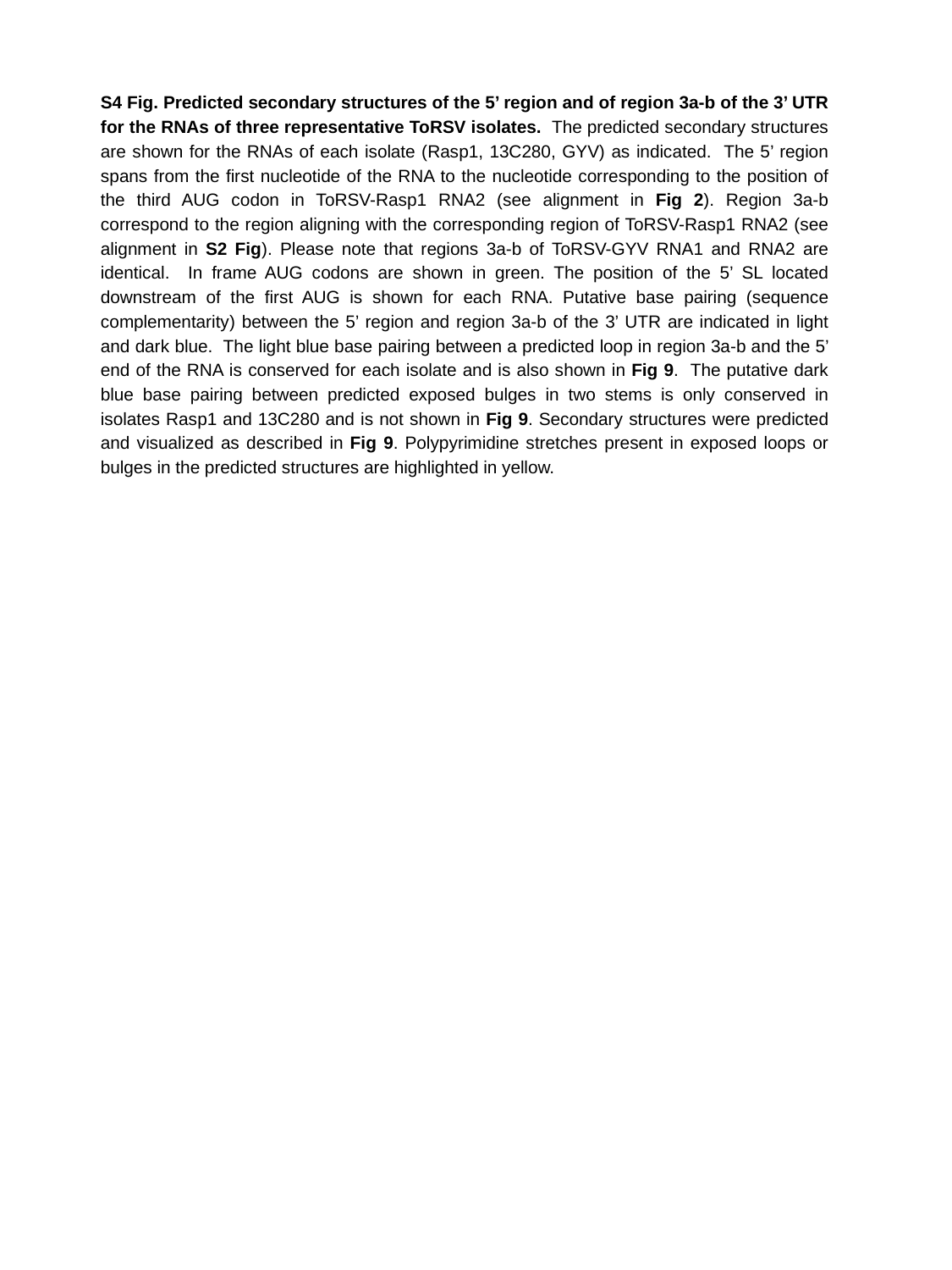

S4 Fig. Predicted secondary structures of the 5’ region and of region 3a-b of the 3’ UTR for the RNAs of three representative ToRSV isolates. The predicted secondary structures are shown for the RNAs of each isolate (Rasp1, 13C280, GYV) as indicated. The 5’ region spans from the first nucleotide of the RNA to the nucleotide corresponding to the position of the third AUG codon in ToRSV-Rasp1 RNA2 (see alignment in Fig 2). Region 3a-b correspond to the region aligning with the corresponding region of ToRSV-Rasp1 RNA2 (see alignment in S2 Fig). Please note that regions 3a-b of ToRSV-GYV RNA1 and RNA2 are identical. In frame AUG codons are shown in green. The position of the 5’ SL located downstream of the first AUG is shown for each RNA. Putative base pairing (sequence complementarity) between the 5’ region and region 3a-b of the 3’ UTR are indicated in light and dark blue. The light blue base pairing between a predicted loop in region 3a-b and the 5’ end of the RNA is conserved for each isolate and is also shown in Fig 9. The putative dark blue base pairing between predicted exposed bulges in two stems is only conserved in isolates Rasp1 and 13C280 and is not shown in Fig 9. Secondary structures were predicted and visualized as described in Fig 9. Polypyrimidine stretches present in exposed loops or bulges in the predicted structures are highlighted in yellow.

## Slide 2
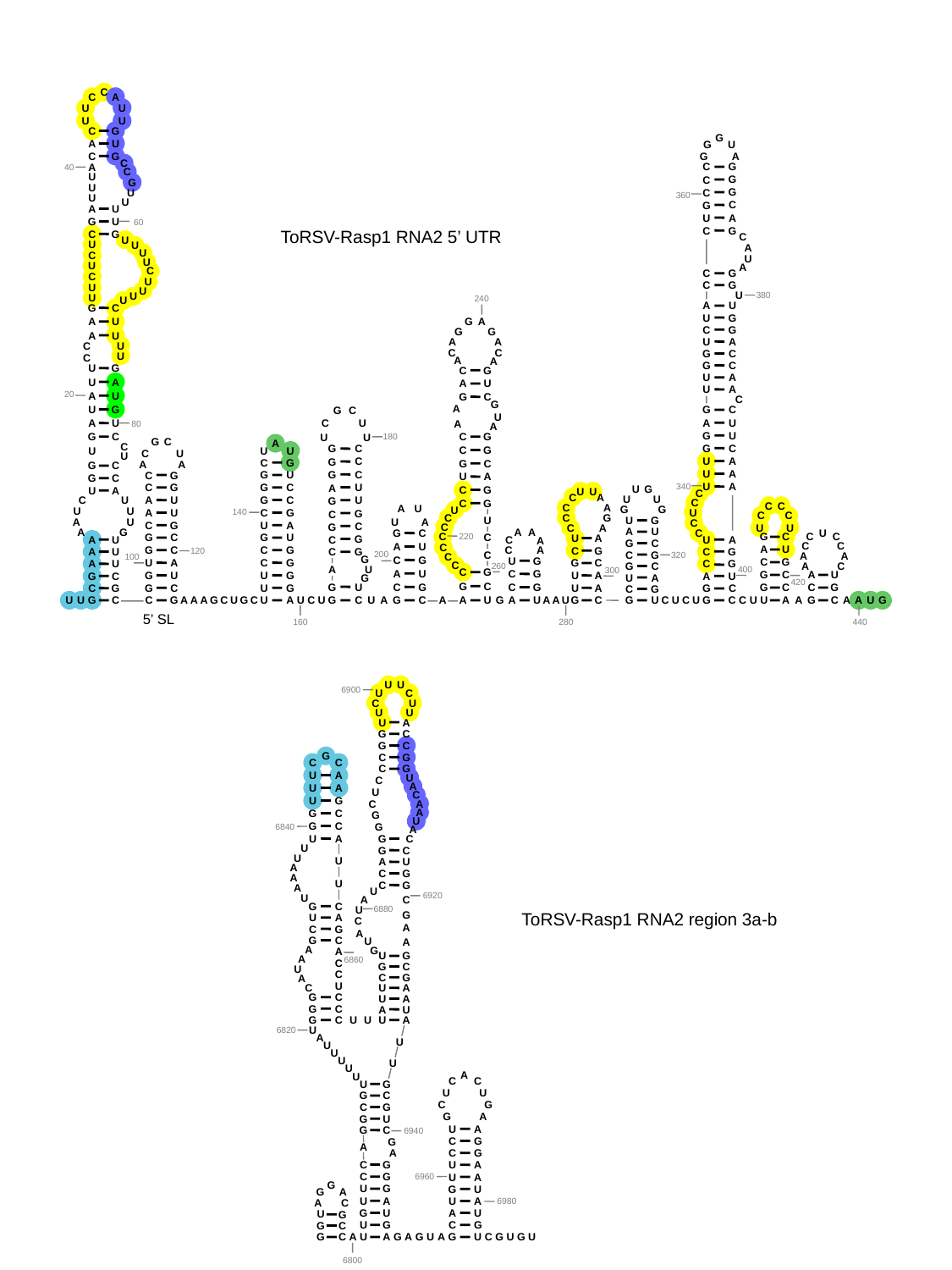

C
C
A
U
U
U
U
C
G
G
A
U
G
U
A
C
G
G
C
C
G
40
A
C
U
G
C
G
U
G
C
U
360
U
U
C
G
A
U
A
U
G
U
60
C
G
C
G
ToRSV-Rasp1 RNA2 5’ UTR
C
U
U
U
A
U
C
U
U
U
A
C
C
G
C
U
C
G
U
U
380
U
U
U
240
U
A
U
G
C
U
G
G
A
A
U
C
G
G
G
A
U
U
A
A
A
C
U
C
C
G
C
U
C
A
A
G
C
U
G
C
G
U
A
U
A
A
U
U
A
20
A
U
G
C
C
G
A
U
G
G
C
G
C
U
A
U
C
U
A
U
80
A
A
G
U
180
C
G
G
C
U
U
G
C
A
C
G
C
G
C
G
C
U
U
U
C
U
U
U
A
C
G
C
G
C
G
A
A
G
C
U
A
C
G
G
U
C
G
A
U
G
C
340
U
A
U
G
C
C
G
A
U
G
G
C
U
A
U
U
C
C
A
U
U
C
U
G
C
A
U
U
G
C
C
G
C
C
C
A
A
U
G
G
U
U
U
140
U
C
G
A
U
G
C
C
C
C
G
C
U
G
U
A
U
U
A
C
U
A
C
G
C
C
G
U
U
C
A
A
U
A
A
G
A
C
U
G
C
C
C
220
C
G
C
C
G
U
G
C
U
A
G
A
U
U
A
C
C
A
G
C
C
C
A
U
C
A
U
C
G
G
C
C
A
120
C
G
A
U
C
G
C
G
200
C
G
C
320
A
A
100
C
G
C
G
U
G
C
G
C
G
U
A
G
C
A
U
C
G
C
G
C
260
A
C
U
A
400
300
C
G
A
U
C
G
G
C
A
U
U
G
G
U
U
A
G
C
A
U
U
A
G
420
G
C
G
U
C
G
C
G
G
C
C
G
U
G
G
C
U
A
C
G
G
C
C
G
U
U
G
C
C
G
A
A
A
G
C
U
G
C
U
A
U
C
U
G
C
U
A
G
C
A
A
U
G
A
U
A
A
U
G
C
G
U
C
U
C
U
G
C
C
U
U
A
A
G
C
A
A
U
G
 5’ SL
160
280
440
U
U
6900
U
C
C
U
U
U
U
A
G
C
G
C
G
C
G
C
C
C
G
U
A
U
C
A
U
A
U
C
U
G
A
C
A
G
C
G
U
G
C
6840
G
A
U
A
G
C
U
G
C
U
U
A
U
A
C
G
A
U
C
G
A
U
6920
U
A
C
G
C
6880
U
G
U
A
C
A
C
G
A
G
C
U
A
A
G
A
U
G
6860
A
C
G
C
U
C
C
G
A
U
C
U
A
G
C
U
A
G
C
A
U
G
C
U
U
U
A
U
6820
A
U
U
U
U
U
U
A
U
C
C
U
G
U
U
G
C
C
G
C
G
G
A
G
U
U
A
G
C
6940
C
G
G
A
C
G
A
C
G
U
A
6960
C
G
U
A
G
U
G
G
U
G
A
U
A
U
A
6980
A
C
G
U
A
U
U
G
U
G
C
G
G
C
G
C
A
U
A
G
A
G
U
A
G
U
C
G
U
G
U
6800
ToRSV-Rasp1 RNA2 region 3a-b

## Slide 3
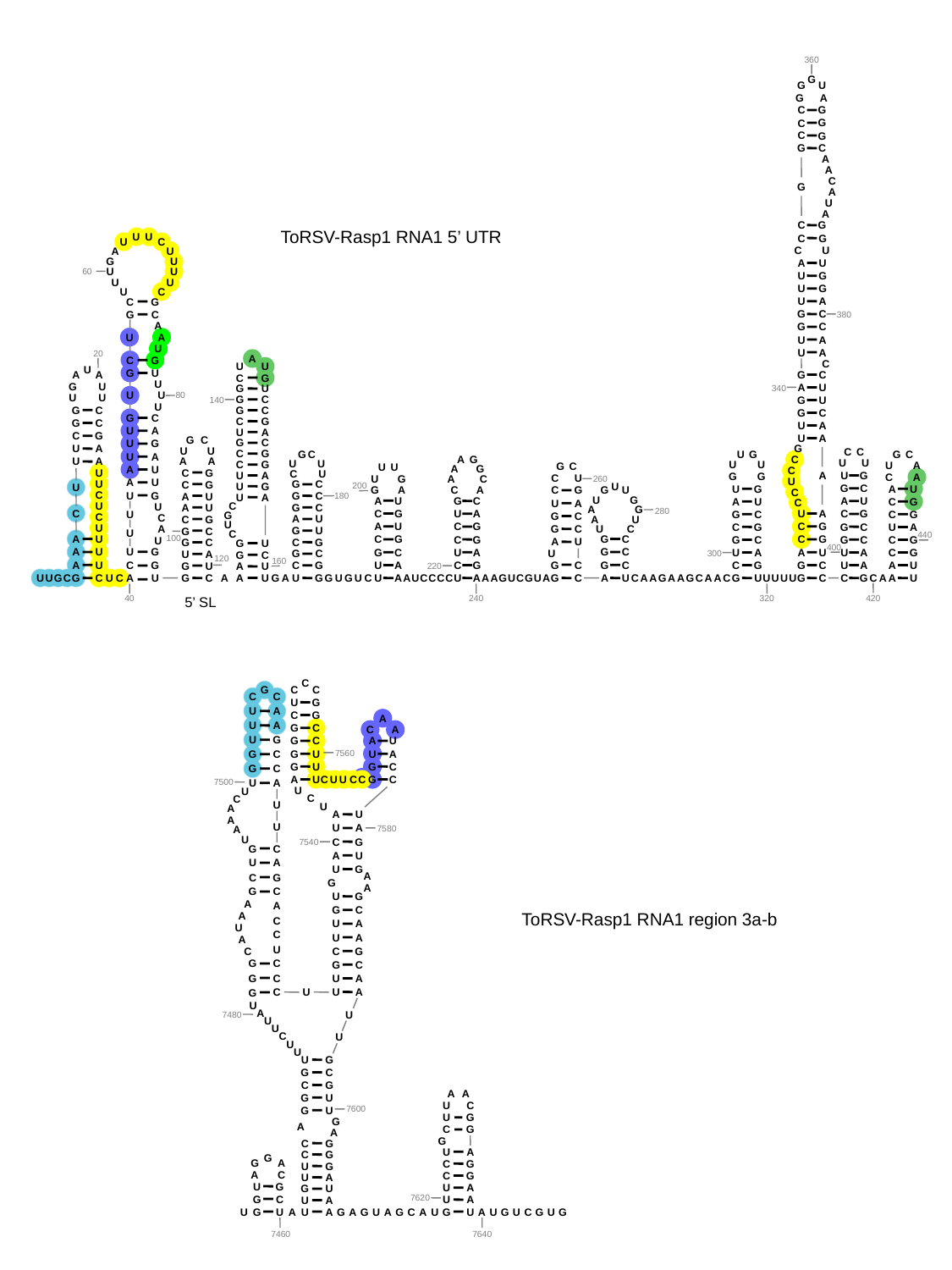

360
G
G
U
G
A
C
G
G
C
C
G
G
C
A
A
C
G
A
U
A
C
G
U
U
C
G
U
C
C
U
A
U
G
U
A
U
U
U
60
U
G
U
U
U
G
U
C
U
A
C
G
G
C
380
G
C
A
G
C
A
U
U
A
U
U
A
20
A
C
G
C
U
U
U
G
U
A
A
G
C
C
G
U
G
U
A
U
340
G
U
80
U
U
U
U
G
C
140
G
U
U
G
C
G
C
G
C
G
C
C
G
G
C
U
A
A
U
U
A
C
G
U
A
G
C
G
C
G
U
U
A
G
U
U
C
C
C
G
U
G
G
C
G
C
A
U
C
A
G
A
A
U
A
U
U
U
U
C
G
U
U
U
A
G
C
U
U
A
G
U
A
C
U
C
G
C
U
U
A
U
G
A
G
G
C
A
C
U
U
G
260
A
C
U
U
A
U
G
C
C
G
200
U
G
U
U
G
C
U
G
A
U
G
A
C
A
C
G
G
U
C
C
U
G
180
G
C
A
U
U
A
U
G
A
U
G
C
A
U
A
U
C
G
C
U
A
U
C
U
G
C
A
U
A
G
280
C
G
U
A
C
U
A
C
G
U
G
C
C
G
G
G
C
C
C
A
U
C
G
A
U
U
A
U
C
G
C
G
G
C
C
G
U
A
U
G
C
U
C
A
G
U
G
C
U
C
440
100
A
U
G
C
G
C
G
C
C
G
G
C
G
C
C
G
U
A
U
C
G
G
C
G
U
400
U
G
A
U
G
C
G
C
A
U
U
A
U
A
U
A
C
G
U
300
G
C
U
A
G
C
120
160
G
C
C
G
A
U
G
C
U
G
C
A
C
G
U
A
C
G
A
U
C
G
G
U
220
A
U
U
U
G
C
G
C
U
C
A
U
G
C
A
A
U
G
A
U
G
G
U
G
U
C
U
A
A
U
C
C
C
C
U
A
A
A
G
U
C
G
U
A
G
C
A
U
C
A
A
G
A
A
G
C
A
A
C
G
U
U
U
U
U
G
C
C
G
C
A
A
U
420
40
240
320
ToRSV-Rasp1 RNA1 5’ UTR
 5’ SL
C
C
C
G
C
C
U
G
U
A
C
G
A
U
A
G
C
C
A
U
G
G
C
A
U
7560
G
U
U
A
G
C
G
U
G
C
G
C
A
U
C
U
U
C
C
G
C
7500
U
A
U
U
C
C
U
U
A
A
U
A
U
U
A
7580
A
U
C
G
7540
G
C
A
U
U
A
U
G
A
C
G
G
A
G
C
U
G
A
A
G
C
A
C
U
A
U
C
U
A
A
U
C
G
C
G
C
G
C
C
G
U
A
A
C
U
U
G
U
A
7480
U
U
U
C
U
U
U
U
G
G
C
C
G
A
A
G
U
U
C
7600
G
U
U
G
G
A
C
G
A
G
C
G
U
A
C
G
G
G
A
C
G
U
G
A
C
C
G
U
A
U
G
U
A
G
U
7620
G
C
U
A
U
A
G
U
A
U
A
G
A
G
U
A
G
C
A
U
G
U
A
U
G
U
C
G
U
G
U
7460
7640
ToRSV-Rasp1 RNA1 region 3a-b

## Slide 4
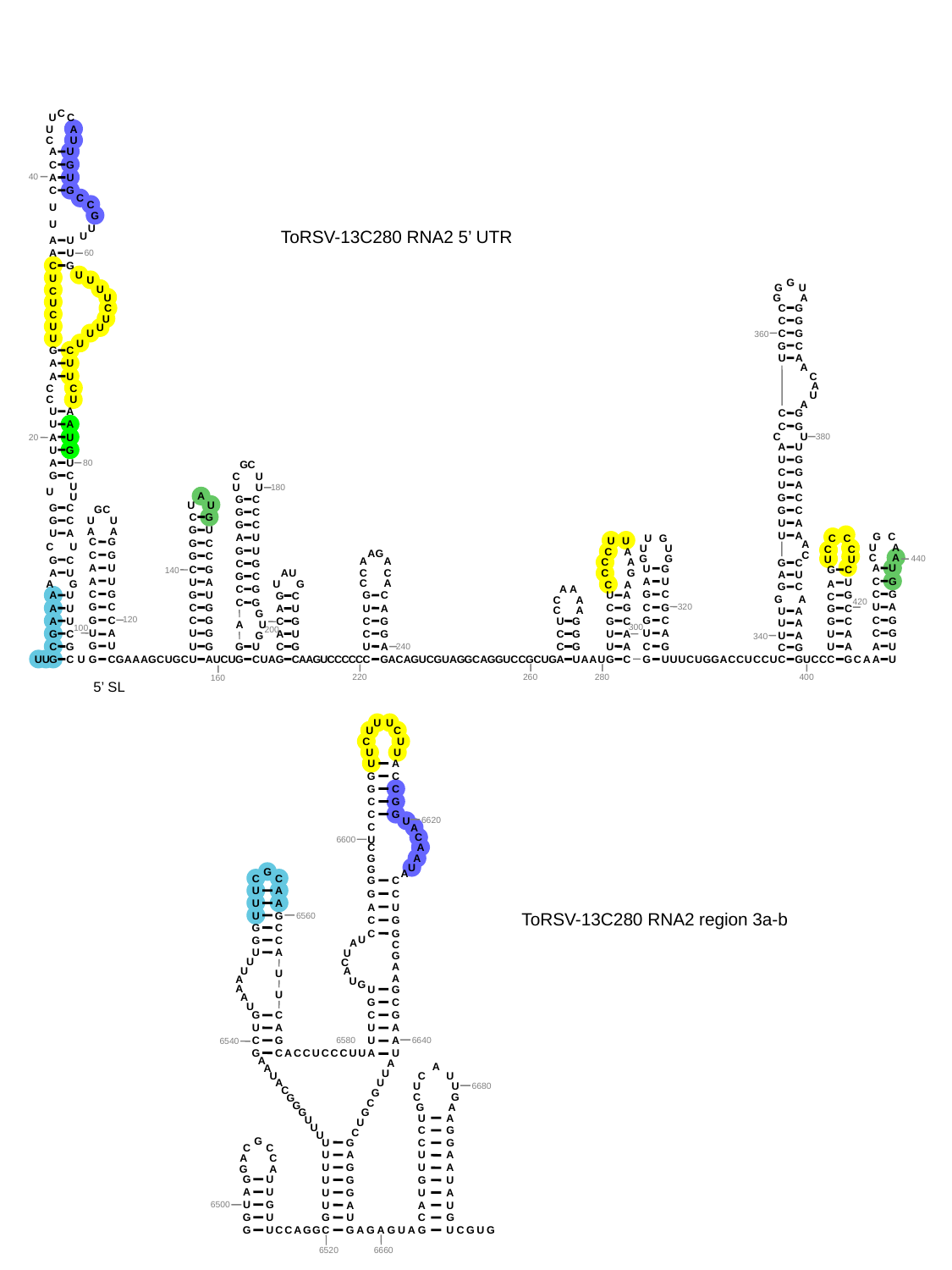

C
U
C
U
A
C
U
A
U
C
G
40
A
U
C
G
C
C
U
G
U
U
U
A
U
A
U
60
C
G
U
U
U
G
G
U
U
C
U
G
A
U
C
G
C
C
U
C
G
U
U
C
G
U
360
U
U
G
C
G
C
U
A
A
U
A
C
A
U
A
C
C
U
C
U
A
U
A
C
G
U
A
C
G
U
C
380
A
U
20
A
U
U
G
U
G
80
A
U
G
C
C
G
G
C
C
U
U
A
U
180
U
U
U
A
U
G
C
G
C
U
U
G
C
G
C
G
C
G
C
C
G
U
U
G
C
U
A
G
C
G
U
A
A
U
A
U
A
G
C
A
U
U
G
C
C
U
U
C
G
G
C
A
C
U
U
A
U
U
C
C
G
U
C
A
A
G
C
C
G
G
C
C
A
440
G
G
U
U
G
C
A
A
C
A
G
C
C
G
A
U
A
U
U
G
C
G
G
C
140
A
U
A
U
C
C
C
G
A
U
G
C
A
U
C
G
A
U
U
A
U
C
A
A
A
G
U
G
C
A
G
C
A
A
C
G
C
G
C
G
G
C
G
U
U
A
G
A
U
G
C
G
C
C
G
A
C
A
420
C
G
320
G
C
U
A
C
G
C
G
G
C
C
A
U
U
A
A
U
G
C
A
U
A
G
120
G
C
C
G
C
G
G
C
C
G
C
A
U
C
G
U
G
C
G
G
U
A
A
U
300
100
200
U
A
U
G
C
G
U
A
A
U
A
G
C
C
G
C
G
A
U
U
U
A
G
340
G
U
240
A
U
U
C
G
U
G
A
A
C
G
U
A
C
G
C
G
U
G
U
C
G
U
U
G
C
U
G
C
G
A
A
A
G
C
U
G
C
U
A
U
C
U
G
C
U
A
G
C
A
A
G
U
C
C
C
C
C
C
G
A
C
A
G
U
C
G
U
A
G
G
C
A
G
G
U
C
C
G
C
U
G
A
U
A
A
U
G
C
G
U
U
U
C
U
G
G
A
C
C
U
C
C
U
C
G
U
C
C
C
G
C
A
A
U
260
280
220
400
160
ToRSV-13C280 RNA2 5’ UTR
 5’ SL
U
U
U
C
C
U
U
U
U
A
G
C
G
C
C
G
C
G
6620
U
C
A
C
6600
U
A
C
A
G
U
G
G
A
C
C
G
C
U
A
G
C
U
A
A
U
U
G
6560
C
G
G
C
C
G
U
G
C
A
C
U
A
U
G
U
C
A
U
A
U
A
A
U
G
A
U
G
U
A
G
C
U
C
G
G
C
U
A
U
A
6580
6640
U
A
C
G
6540
G
C
A
C
C
U
C
C
C
U
U
A
U
A
A
A
A
U
U
C
U
A
U
6680
U
U
C
G
C
G
G
C
G
G
A
G
G
U
A
U
U
U
C
G
C
U
G
U
G
C
G
C
C
U
A
U
A
A
C
U
A
U
G
G
A
G
U
U
G
G
U
A
U
U
G
U
A
6500
U
G
U
A
A
U
G
U
C
G
G
U
G
U
C
C
A
G
G
C
G
A
G
A
G
U
A
G
U
C
G
U
G
6660
6520
ToRSV-13C280 RNA2 region 3a-b

## Slide 5
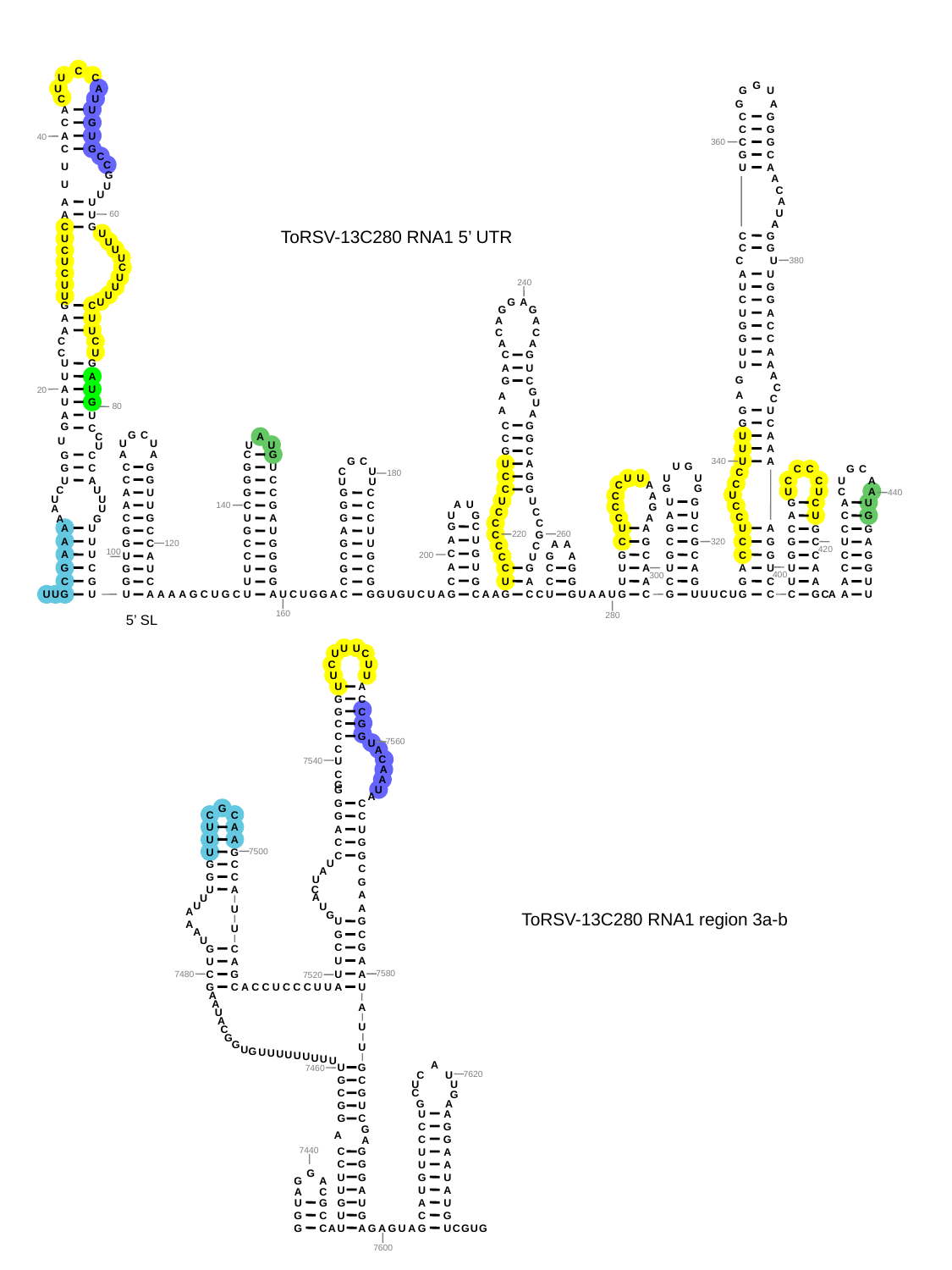

C
U
C
G
U
A
G
U
C
U
G
A
A
U
C
G
C
G
C
G
A
U
40
C
G
360
C
G
G
C
C
C
U
U
A
G
A
U
U
C
U
A
A
U
U
60
A
U
A
C
G
U
C
G
U
U
C
G
U
C
U
380
C
U
U
C
C
A
U
U
240
U
U
G
U
U
U
C
G
U
G
A
G
C
G
G
U
A
A
U
A
A
G
C
A
U
C
C
G
C
C
C
A
A
U
A
C
U
C
G
U
G
U
A
A
U
A
U
A
G
G
C
C
A
U
20
G
A
A
C
U
G
U
80
G
U
A
A
A
U
G
C
C
G
G
C
G
C
U
A
A
C
C
G
U
U
U
U
U
U
U
A
G
C
A
A
C
G
G
C
G
C
U
A
340
U
A
U
G
C
G
G
U
G
C
C
C
G
C
C
U
C
180
C
G
U
U
U
U
C
G
G
C
U
A
C
C
U
A
U
U
C
C
A
G
G
C
G
C
U
U
U
C
A
440
A
U
G
C
G
C
U
C
A
U
U
U
U
U
G
G
C
A
U
A
U
A
U
140
C
G
G
C
C
C
G
A
U
C
C
A
U
A
U
C
G
U
G
C
C
G
U
A
G
C
C
A
A
G
C
C
G
C
A
U
U
A
G
C
U
A
C
G
C
G
G
C
G
U
A
U
260
220
C
G
A
U
A
U
320
C
G
C
G
C
G
G
C
U
A
120
G
C
C
G
G
U
A
A
C
C
420
100
C
G
A
U
200
G
C
G
C
C
G
G
C
C
G
G
A
U
A
C
G
C
G
C
U
A
U
U
A
G
C
C
G
C
G
U
A
A
U
U
A
C
G
G
U
U
G
G
C
400
300
C
G
U
A
C
G
U
A
C
G
C
G
G
C
U
A
A
U
G
C
U
G
C
G
U
U
G
U
U
A
A
A
A
G
C
U
G
C
U
A
U
C
U
G
G
A
C
G
G
U
G
U
C
U
A
G
C
A
A
G
C
C
U
G
U
A
A
U
G
C
G
U
U
U
C
U
G
C
C
G
C
A
A
U
160
280
ToRSV-13C280 RNA1 5’ UTR
 5’ SL
U
U
U
C
C
U
U
U
U
A
G
C
G
C
C
G
C
G
7560
U
C
A
C
7540
U
A
C
A
G
U
G
A
G
C
G
C
C
G
C
U
A
U
A
U
A
C
G
7500
U
G
C
G
U
G
C
C
A
G
C
U
G
C
U
A
A
A
U
U
U
A
U
A
G
U
G
A
U
A
G
C
U
C
G
G
C
U
A
U
A
7580
U
A
C
G
7480
7520
G
C
A
C
C
U
C
C
C
U
U
A
U
A
A
A
U
A
U
C
G
G
U
U
G
U
U
U
U
U
U
U
U
U
A
U
G
7460
7620
C
U
G
C
U
U
C
G
C
G
G
A
G
U
U
A
G
C
C
G
G
A
C
G
A
7440
C
G
U
A
G
C
U
A
G
G
U
G
U
G
A
A
U
U
A
A
C
U
G
U
G
A
U
G
U
G
C
C
G
G
C
A
U
A
G
A
G
U
A
G
U
C
G
U
G
7600
ToRSV-13C280 RNA1 region 3a-b

## Slide 6
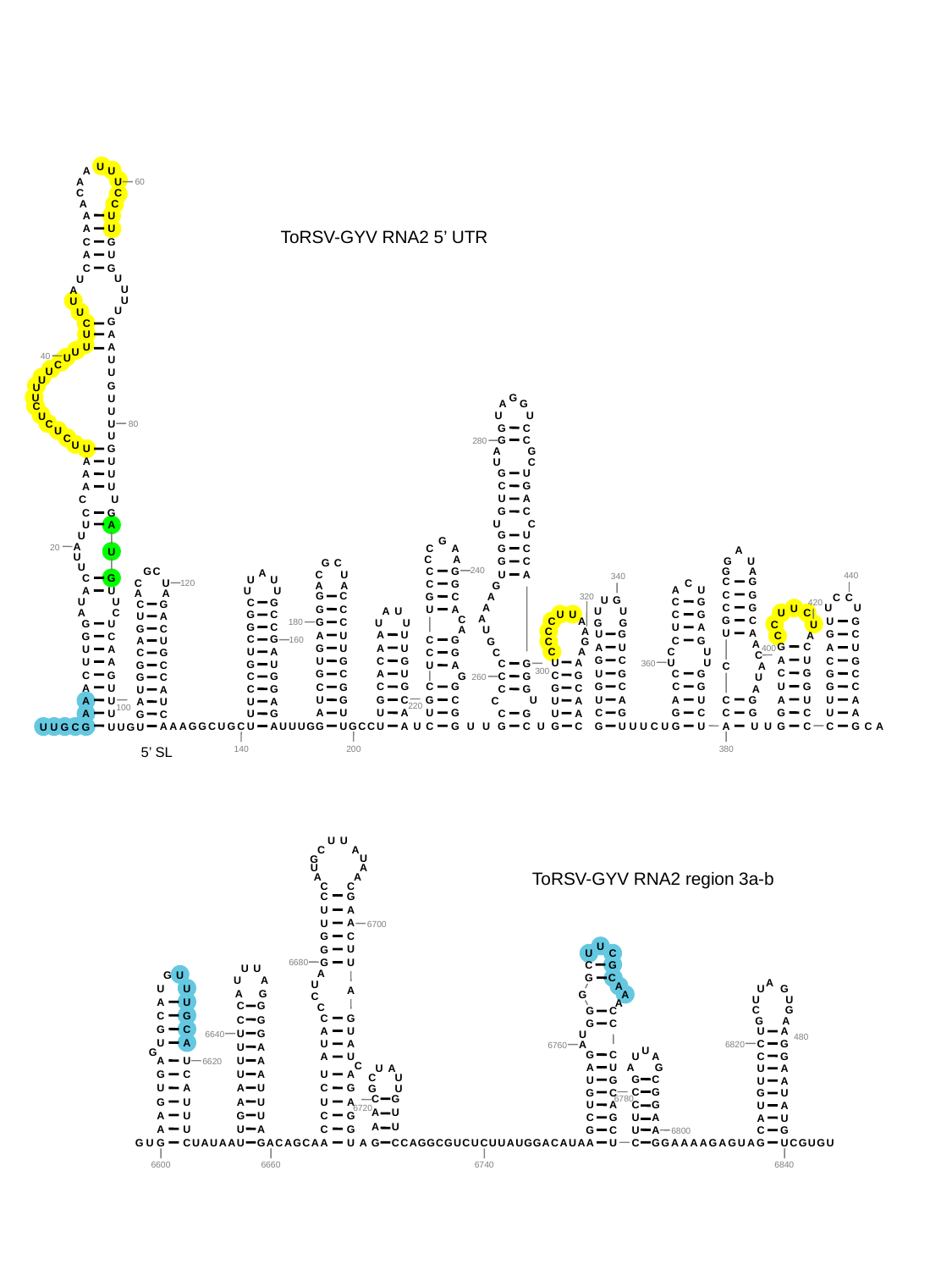

U
A
U
A
U
60
C
C
A
C
A
U
A
U
C
G
A
U
C
G
U
U
U
A
U
U
U
U
G
C
U
A
U
A
U
40
U
U
C
U
U
U
G
U
U
G
U
A
G
C
U
U
U
U
80
U
C
G
C
U
U
C
G
C
280
U
U
G
A
G
A
U
U
C
G
U
A
U
C
G
A
U
U
A
C
U
G
C
C
G
U
C
U
A
G
U
U
G
A
20
C
A
G
C
A
U
U
C
A
G
U
G
C
G
C
U
240
C
G
G
A
G
C
A
C
U
U
A
440
340
C
G
U
U
C
G
C
U
120
C
C
G
A
A
G
A
U
A
U
U
U
A
A
C
G
G
C
320
G
C
A
C
C
U
G
U
U
C
G
420
C
G
C
G
C
G
A
U
U
U
G
C
U
A
U
U
A
U
A
C
U
C
U
U
C
G
G
C
U
A
A
C
G
C
C
A
U
G
180
G
C
G
G
U
U
G
U
C
U
G
C
U
A
G
C
A
U
C
A
U
A
U
G
G
C
A
U
A
U
C
A
G
C
160
C
G
C
G
C
G
A
U
G
C
G
A
G
C
A
U
A
U
A
U
G
U
400
U
A
C
U
U
A
C
A
C
C
G
C
G
C
A
U
G
C
C
G
C
G
U
G
U
A
U
A
U
U
C
G
360
G
U
G
C
U
A
A
C
300
C
G
U
G
C
G
G
C
A
U
G
C
C
G
C
G
G
C
G
260
C
G
G
C
U
U
G
C
G
G
C
C
G
G
C
C
G
C
G
G
C
A
U
A
C
G
C
G
U
A
A
U
C
G
G
C
U
A
A
U
U
A
G
C
U
G
U
U
A
C
A
U
U
A
A
U
220
100
G
C
C
G
G
C
C
G
U
A
U
A
U
G
A
U
C
G
U
A
A
U
U
G
G
C
U
G
G
U
G
C
C
U
A
U
C
G
U
U
G
C
U
G
C
G
U
U
U
C
U
G
U
A
U
U
G
C
C
G
C
A
A
A
A
G
G
C
U
G
C
U
A
U
U
U
U
G
C
G
U
U
G
U
200
380
140
ToRSV-GYV RNA2 5’ UTR
 5’ SL
U
U
A
C
U
G
A
U
A
A
C
C
C
G
A
U
A
U
6700
C
G
U
U
G
U
C
G
U
6680
C
G
U
U
A
G
U
G
C
U
A
A
U
A
U
U
U
G
A
A
G
G
A
C
U
U
A
U
A
C
G
C
C
G
G
C
C
G
C
G
C
G
G
A
G
C
G
C
A
U
U
A
U
G
U
6640
480
U
A
C
G
U
A
6820
A
6760
U
A
U
G
G
C
C
G
U
A
A
U
A
U
U
A
6620
C
A
U
A
G
U
A
U
A
G
C
U
A
U
A
C
U
G
C
U
G
U
A
U
A
A
U
C
G
G
U
C
G
G
C
G
U
6780
C
G
G
U
A
U
U
A
C
G
U
A
U
A
6720
A
U
A
U
G
U
C
G
U
A
C
G
A
U
A
U
A
U
U
A
C
G
U
A
G
C
C
G
6800
G
U
G
C
U
A
U
A
A
U
G
A
C
A
G
C
A
A
U
A
G
C
C
A
G
G
C
G
U
C
U
C
U
U
A
U
G
G
A
C
A
U
A
A
U
C
G
G
A
A
A
A
G
A
G
U
A
G
U
C
G
U
G
U
6600
6660
6740
6840
ToRSV-GYV RNA2 region 3a-b

## Slide 7
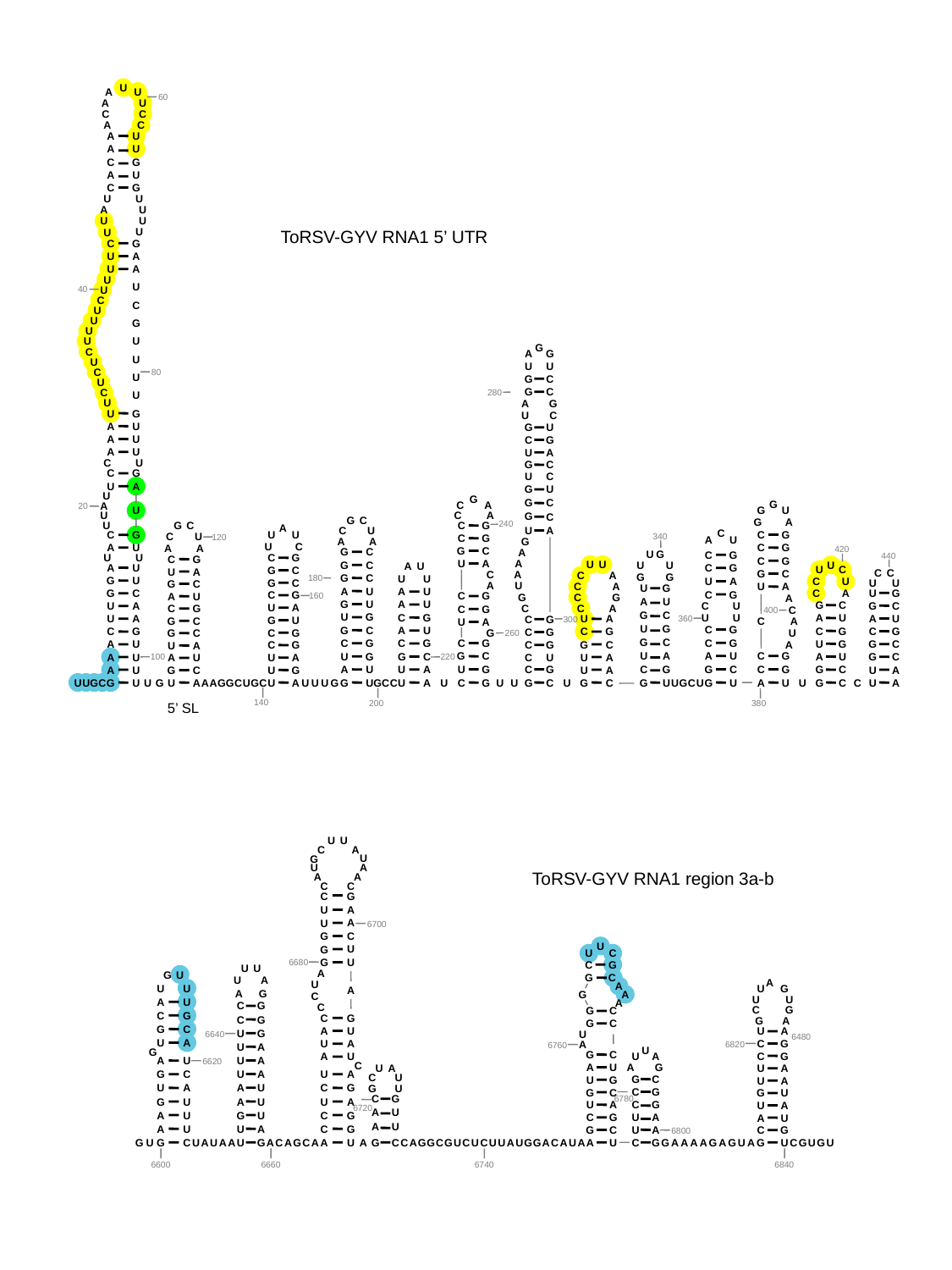

U
A
U
60
A
U
C
C
A
C
A
U
A
U
C
G
A
U
C
G
U
U
U
A
U
U
U
U
C
G
U
A
U
A
U
U
40
U
C
C
U
U
G
U
U
U
G
C
A
G
U
U
U
U
80
C
U
G
C
U
G
C
280
C
U
U
A
G
U
G
U
C
A
U
G
U
A
U
C
G
A
U
U
A
C
U
G
C
C
G
U
C
U
A
G
U
U
G
G
C
G
C
A
A
20
G
U
U
C
A
U
G
C
G
C
G
A
240
U
G
C
C
G
A
U
A
C
U
C
C
G
C
G
U
U
340
C
U
120
C
G
A
U
A
A
G
U
C
A
U
C
G
A
A
420
G
C
G
C
A
U
G
C
G
440
U
U
C
G
C
G
C
G
U
A
A
U
U
G
C
U
U
U
A
U
A
U
C
G
U
C
G
C
U
A
C
C
G
C
C
A
C
A
G
G
180
G
C
U
U
G
U
U
A
C
U
U
U
G
C
G
C
A
U
C
A
U
A
U
G
A
U
A
U
U
G
C
A
G
C
C
G
C
G
160
C
G
A
U
G
C
G
A
A
U
G
U
A
U
G
C
G
C
U
A
C
U
U
A
C
A
C
C
G
C
G
C
400
G
C
U
G
C
G
U
U
A
U
360
A
U
U
A
300
U
A
C
G
G
U
G
C
C
A
U
A
U
G
C
G
G
C
A
U
C
G
C
G
C
G
C
G
C
G
G
C
G
260
U
G
C
G
C
C
G
C
G
C
G
C
G
U
G
G
C
A
U
G
C
A
C
G
C
G
U
A
U
A
C
G
A
U
100
G
C
U
G
G
C
A
U
220
G
C
A
U
U
A
C
U
U
A
A
U
C
G
C
G
C
G
G
C
U
G
A
U
U
A
G
C
U
A
A
U
U
A
U
G
G
C
U
U
G
C
G
U
U
G
U
A
A
A
G
G
C
U
G
C
U
A
U
U
U
G
G
U
G
C
C
U
A
U
C
G
U
U
G
C
U
G
C
G
U
U
G
C
U
G
U
A
U
U
G
C
C
U
A
140
200
380
ToRSV-GYV RNA1 5’ UTR
 5’ SL
U
U
A
C
U
G
A
U
A
A
C
C
C
G
A
U
A
U
6700
C
G
U
U
G
U
C
G
U
6680
C
G
U
U
A
G
U
G
C
U
A
A
U
A
U
U
U
G
A
A
G
G
A
C
U
U
A
U
A
C
G
C
C
G
G
C
C
G
C
G
C
G
G
A
G
C
G
C
A
U
U
A
U
G
U
6640
6480
U
A
C
G
U
A
6820
A
6760
U
A
U
G
G
C
C
G
U
A
A
U
A
U
U
A
6620
C
A
U
A
G
U
A
U
A
G
C
U
A
U
A
C
U
G
C
U
G
U
A
U
A
A
U
C
G
G
U
C
G
G
C
G
U
6780
C
G
G
U
A
U
U
A
C
G
U
A
U
A
6720
A
U
A
U
G
U
C
G
U
A
C
G
A
U
A
U
A
U
U
A
C
G
U
A
G
C
C
G
6800
G
U
G
C
U
A
U
A
A
U
G
A
C
A
G
C
A
A
U
A
G
C
C
A
G
G
C
G
U
C
U
C
U
U
A
U
G
G
A
C
A
U
A
A
U
C
G
G
A
A
A
A
G
A
G
U
A
G
U
C
G
U
G
U
6600
6660
6740
6840
ToRSV-GYV RNA1 region 3a-b
