# Supplementary material for: Mapping of sequences in the 5’ region and 3’ UTR of tomato ringspot virus RNA2 that facilitate cap-independent translation of reporter transcripts in vitro
Source: PLoS One. 2021 Apr 9;16(4):e0249928. doi: 10.1371/journal.pone.0249928 (PMC8034749; doi:10.1371/journal.pone.0249928)
Supplement: S5 Fig — The nucleotide sequence of the 3’ UTRs of RNA1 and RNA2 were aligned by Clustal W as implemented in MEGA X. Phylogenetic trees were generated using the maximum likelihood method and the validity of the branches was verified using 1000 bootstraps. For comparison, the deduced amino acid sequence of the Pro-Pol region (encoded by RNA1 and defined as the region between the catalytic cysteine of the protease and the GDD motif of the polymerase) and of the coat protein (encoded by RNA2) were also aligned by Clustal W and phylogenies were produced and tested as above. (PPTX) [file pone.0249928.s007.pptx]

## Slide 1
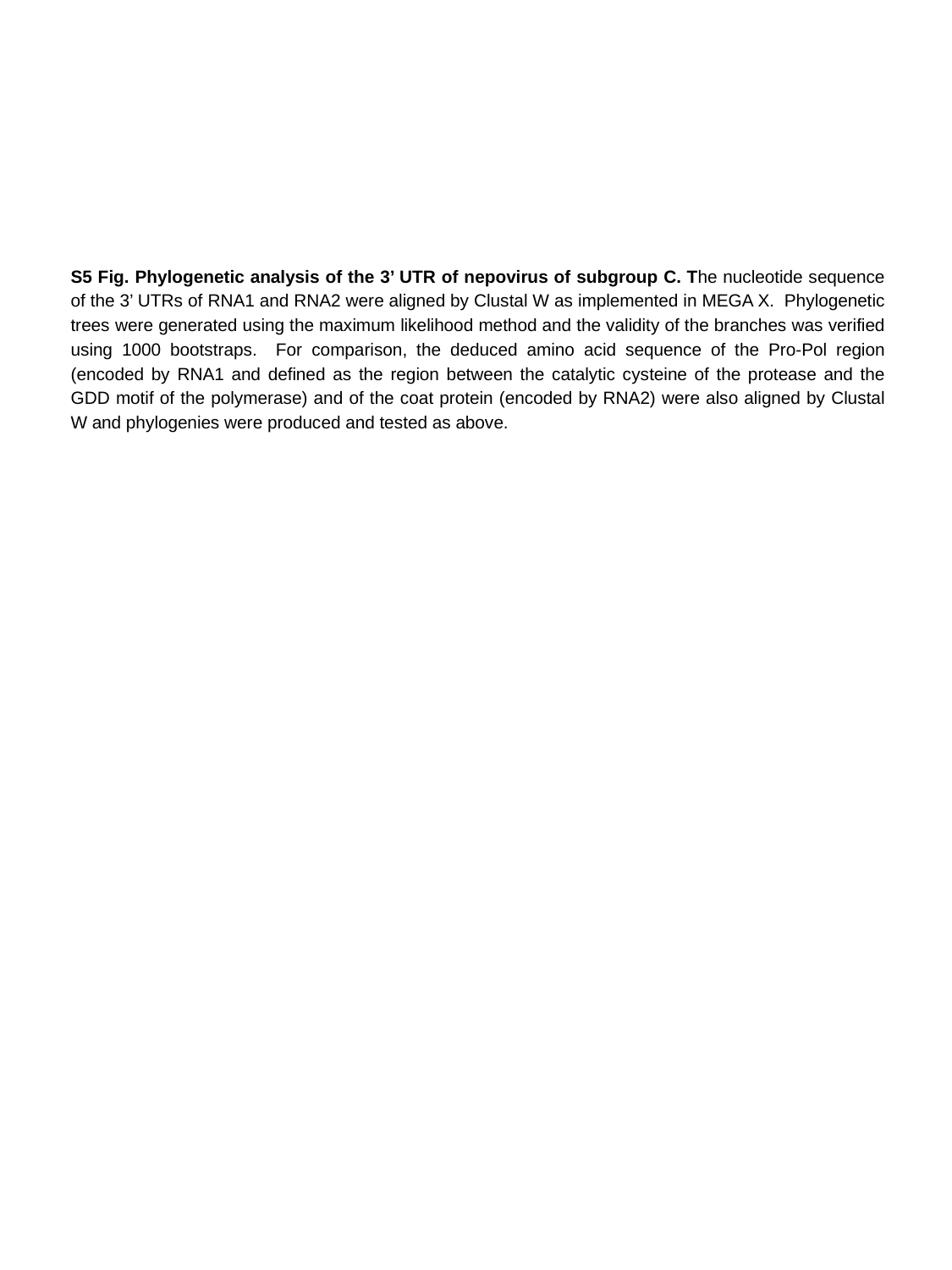

S5 Fig. Phylogenetic analysis of the 3’ UTR of nepovirus of subgroup C. The nucleotide sequence of the 3’ UTRs of RNA1 and RNA2 were aligned by Clustal W as implemented in MEGA X. Phylogenetic trees were generated using the maximum likelihood method and the validity of the branches was verified using 1000 bootstraps. For comparison, the deduced amino acid sequence of the Pro-Pol region (encoded by RNA1 and defined as the region between the catalytic cysteine of the protease and the GDD motif of the polymerase) and of the coat protein (encoded by RNA2) were also aligned by Clustal W and phylogenies were produced and tested as above.

## Slide 2
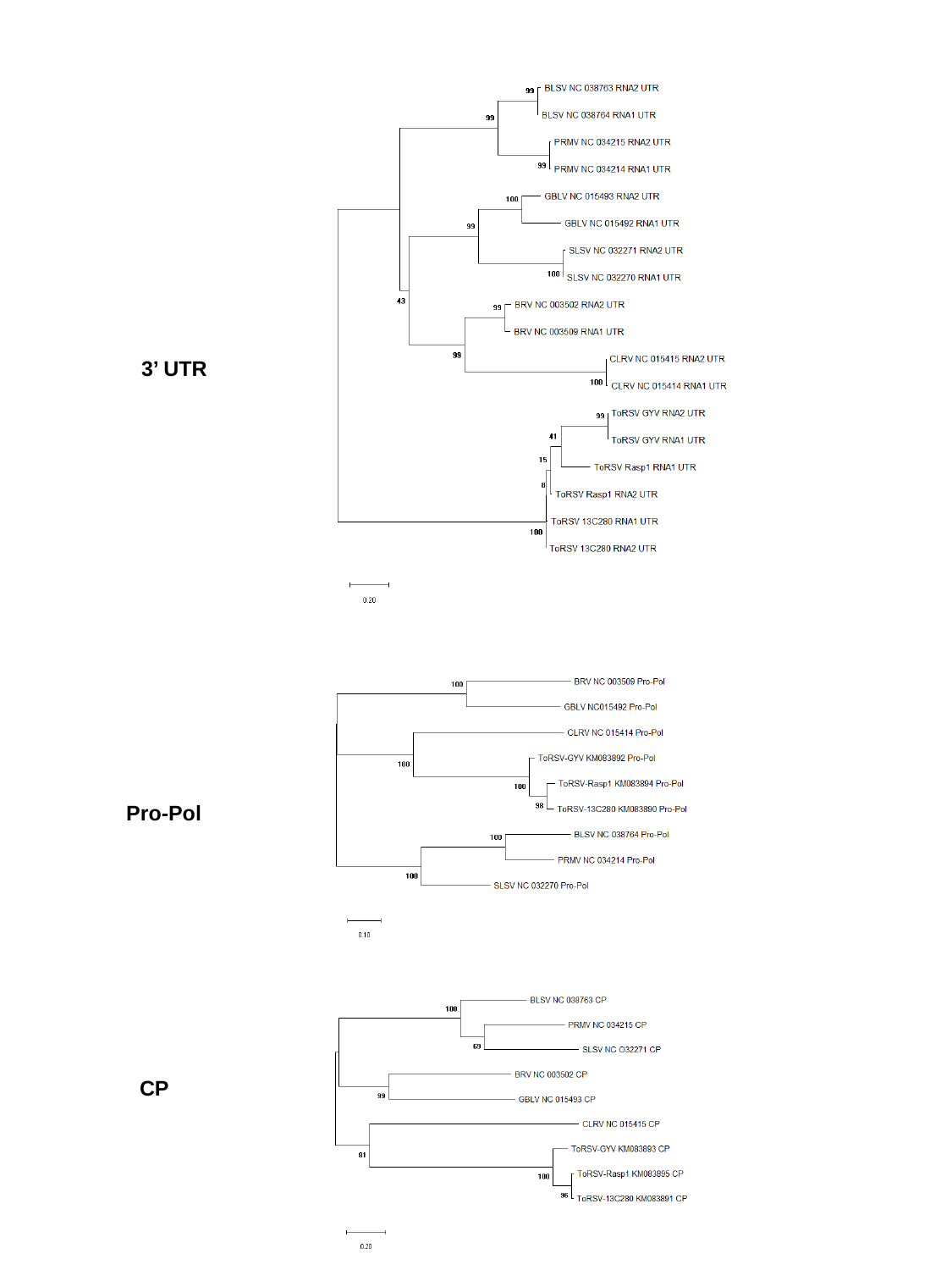

3’ UTR
Pro-Pol
CP
