# Supplementary material for: Mapping of sequences in the 5’ region and 3’ UTR of tomato ringspot virus RNA2 that facilitate cap-independent translation of reporter transcripts in vitro
Source: PLoS One. 2021 Apr 9;16(4):e0249928. doi: 10.1371/journal.pone.0249928 (PMC8034749; doi:10.1371/journal.pone.0249928)
Supplement: S6 Fig — (A) The 5’ region spans from the first 440 nucleotides of the RNA corresponding to the 5’ region shown for ToRSV RNAs in Figs 9and S4. Because the entire predicted secondary structure did not fit in the frame, nts 327–440 are shown above nts 1–327 but would be contiguous in the overall predicted secondary structure. In frame AUG codons are shown in green. The position of a previously identified 5’ SL is shown [42]. The loop of this stem-loop (highlighted in light blue) is involved in an experimentally validated kissing loop interaction with the loop of 3’ SL-1 of the 3’ UTR region A2 shown in (B). Another putative stem-loop located downstream of the first AUG (5’ SL-2) and corresponding to the 5’ SL of ToRSV RNAs is shown. Please note that the 5’ coding region has not been studied for BRV and that the biological relevance of 5’ SL-2 is not known. (B) Regions of the BRV RNA2 3’ UTR previously identified as playing a role in translation of RNA2 [42], see Fig 10A for schematic representation of the position of these regions. The A2 region was shown to be the most critical. It contains two previously identified stem-loops, the first of which (3’ SL-1) is involved in a kissing-loop interaction with the 5’ SL (shown in light blue). Region C2 was shown to play a modest role in translation and includes a proposed alternative base pairing with the 5’ SL (shown in light blue). Secondary structures were predicted and visualized as described in Fig 9. Polypyrimidine stretches present in exposed loops or bulges in the predicted structures are highlighted in yellow. (PPTX) [file pone.0249928.s008.pptx]

## Slide 1
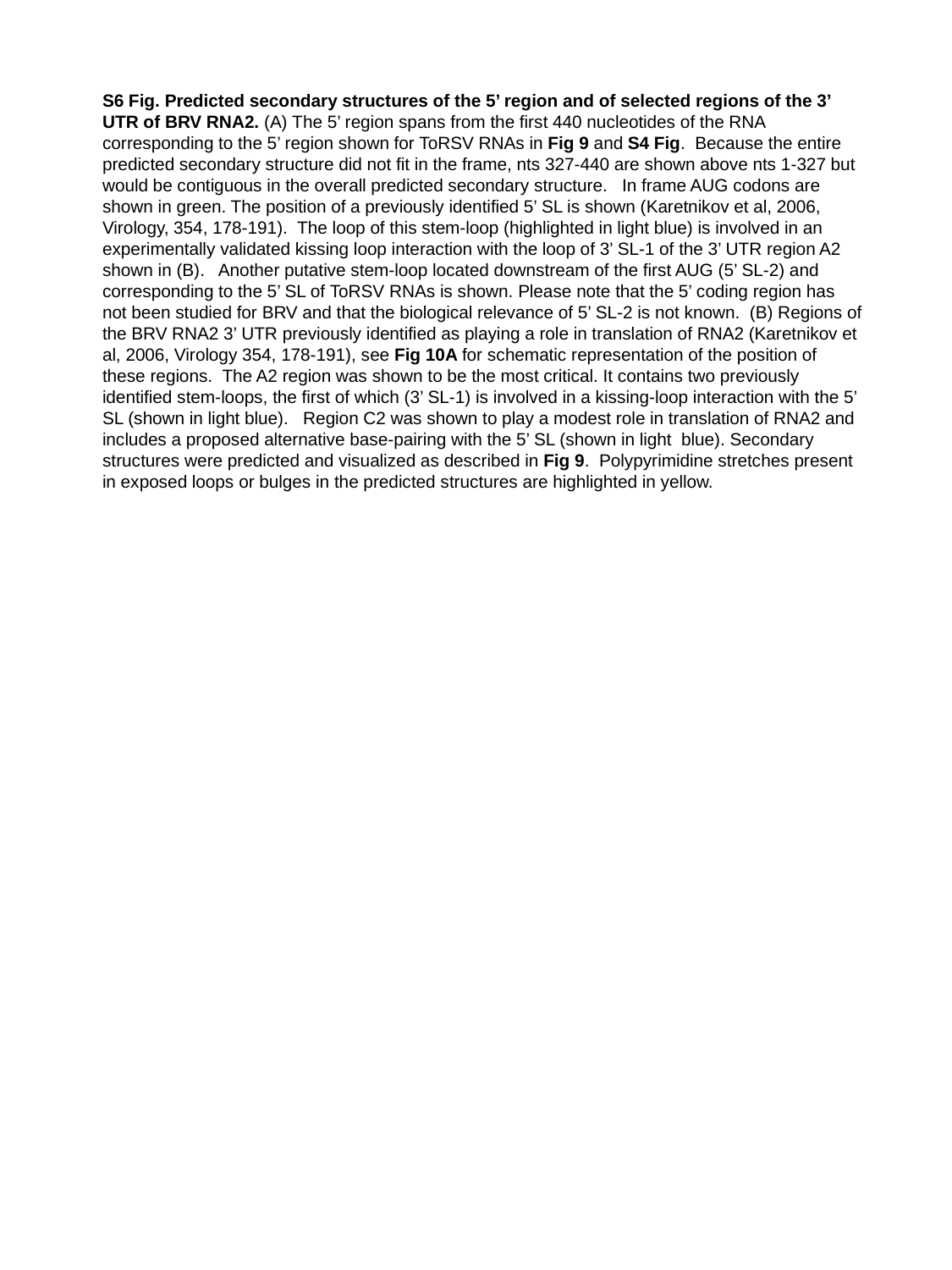

S6 Fig. Predicted secondary structures of the 5’ region and of selected regions of the 3’ UTR of BRV RNA2. (A) The 5’ region spans from the first 440 nucleotides of the RNA corresponding to the 5’ region shown for ToRSV RNAs in Fig 9 and S4 Fig. Because the entire predicted secondary structure did not fit in the frame, nts 327-440 are shown above nts 1-327 but would be contiguous in the overall predicted secondary structure. In frame AUG codons are shown in green. The position of a previously identified 5’ SL is shown (Karetnikov et al, 2006, Virology, 354, 178-191). The loop of this stem-loop (highlighted in light blue) is involved in an experimentally validated kissing loop interaction with the loop of 3’ SL-1 of the 3’ UTR region A2 shown in (B). Another putative stem-loop located downstream of the first AUG (5’ SL-2) and corresponding to the 5’ SL of ToRSV RNAs is shown. Please note that the 5’ coding region has not been studied for BRV and that the biological relevance of 5’ SL-2 is not known. (B) Regions of the BRV RNA2 3’ UTR previously identified as playing a role in translation of RNA2 (Karetnikov et al, 2006, Virology 354, 178-191), see Fig 10A for schematic representation of the position of these regions. The A2 region was shown to be the most critical. It contains two previously identified stem-loops, the first of which (3’ SL-1) is involved in a kissing-loop interaction with the 5’ SL (shown in light blue). Region C2 was shown to play a modest role in translation of RNA2 and includes a proposed alternative base-pairing with the 5’ SL (shown in light blue). Secondary structures were predicted and visualized as described in Fig 9. Polypyrimidine stretches present in exposed loops or bulges in the predicted structures are highlighted in yellow.

## Slide 2
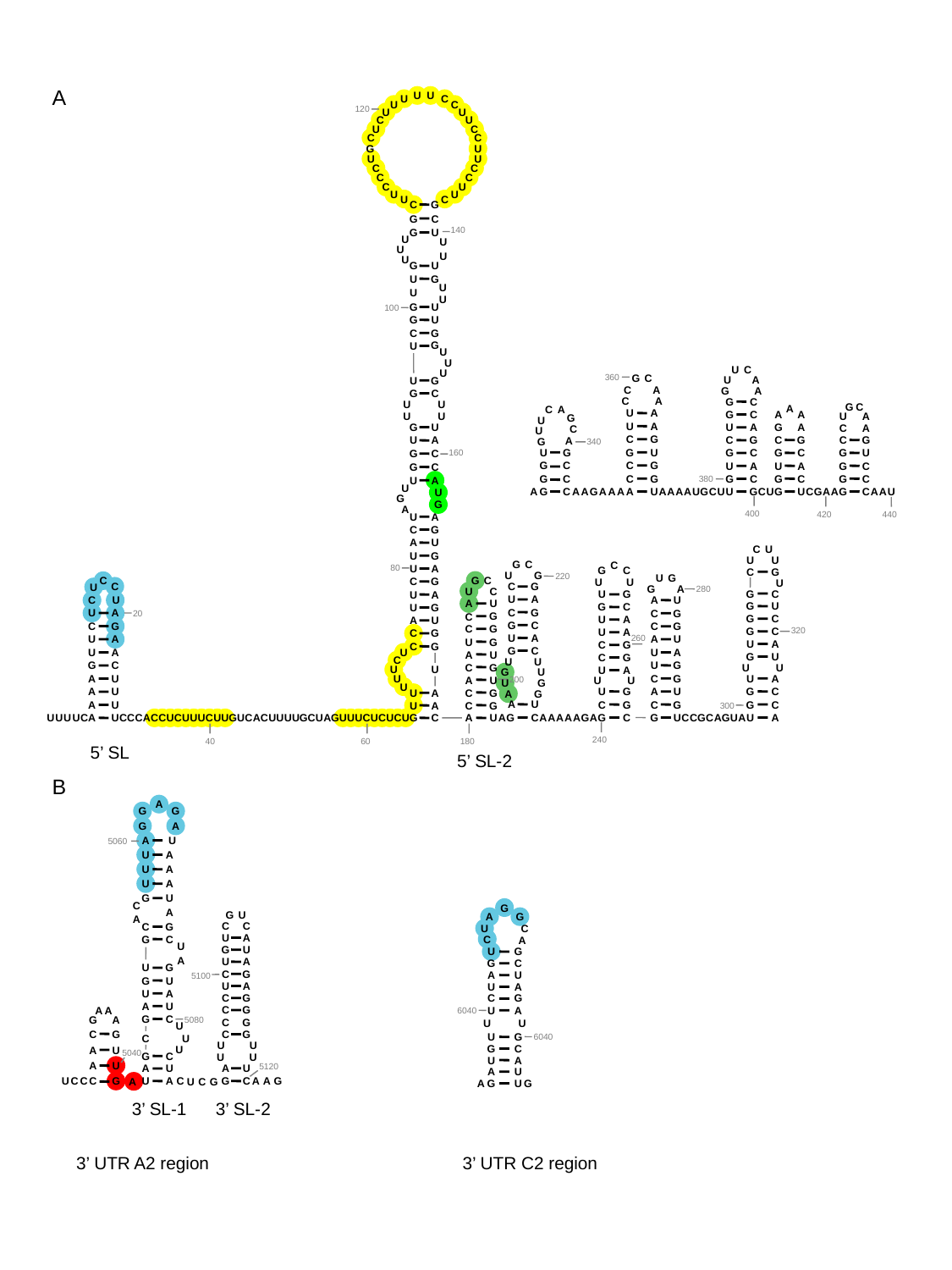

A
U
U
U
C
U
C
120
U
U
C
U
U
C
C
C
G
U
U
U
C
C
C
C
C
U
U
U
U
C
C
G
G
C
140
G
U
U
U
U
U
U
G
U
U
G
U
U
U
G
U
100
G
U
C
G
G
U
U
U
U
C
G
C
U
A
C
A
G
A
C
A
G
C
G
C
A
C
A
U
A
A
A
G
C
U
A
G
U
U
A
G
A
U
A
C
A
C
U
C
G
C
C
G
G
C
G
A
G
U
G
G
U
G
G
U
C
G
C
G
C
C
G
U
G
C
A
U
A
G
C
C
G
G
G
C
C
G
C
A
G
C
A
A
G
A
A
A
A
U
A
A
A
A
U
G
C
U
U
G
C
U
G
U
C
G
A
A
G
C
A
A
U
U
360
U
G
G
C
U
U
U
U
G
U
U
A
340
160
G
C
G
C
380
U
A
U
U
G
G
A
400
420
440
U
A
C
G
A
U
C
U
U
G
U
U
G
C
C
80
U
A
G
C
C
G
U
G
220
U
G
G
C
C
C
G
U
U
U
C
C
G
U
280
G
A
C
U
G
C
U
G
U
A
U
A
A
U
C
U
A
U
G
U
G
C
U
G
C
G
U
A
C
G
20
G
C
G
C
U
A
A
U
G
C
C
G
C
G
C
G
320
G
C
U
A
C
G
260
U
A
U
A
A
U
U
G
U
A
C
G
C
G
G
C
U
A
U
A
U
A
U
G
U
C
G
C
U
U
U
G
G
C
U
U
C
G
U
U
U
A
G
U
U
A
C
G
A
U
U
200
A
U
U
U
U
G
U
G
C
U
G
A
U
A
U
U
A
C
G
A
G
A
U
G
C
C
G
C
G
A
U
300
U
A
C
G
A
G
C
A
A
A
A
A
G
A
G
C
G
U
C
C
G
C
A
G
U
A
U
A
U
U
U
U
C
A
U
C
C
C
A
C
C
U
C
U
U
U
C
U
U
G
U
C
A
C
U
U
U
U
G
C
U
A
G
U
U
U
C
U
C
U
C
U
G
C
A
U
240
180
40
60
5’ SL
5’ SL-2
B
A
G
G
G
A
A
U
5060
U
A
U
A
U
A
G
U
C
G
A
G
U
A
G
A
C
C
C
G
U
C
U
A
C
G
C
A
U
G
U
U
G
A
U
A
G
C
U
G
C
G
A
U
5100
G
U
U
A
U
A
U
A
C
G
C
G
A
U
C
G
6040
U
A
A
A
G
C
5080
G
A
C
G
U
U
U
C
G
G
C
6040
U
G
U
C
U
U
G
C
U
A
U
5040
G
C
U
U
U
A
U
A
A
U
A
U
5120
A
U
U
C
C
G
A
U
C
G
G
C
A
G
C
U
A
C
A
A
G
U
G
3’ SL-1 3’ SL-2
3’ UTR A2 region
3’ UTR C2 region
