# Supplementary material for: Mapping of sequences in the 5’ region and 3’ UTR of tomato ringspot virus RNA2 that facilitate cap-independent translation of reporter transcripts in vitro
Source: PLoS One. 2021 Apr 9;16(4):e0249928. doi: 10.1371/journal.pone.0249928 (PMC8034749; doi:10.1371/journal.pone.0249928)
Supplement: S1 Raw images — (PDF) [file pone.0249928.s009.pdf]

Raw image Fig. 3B
Top panel (radiolabelled)

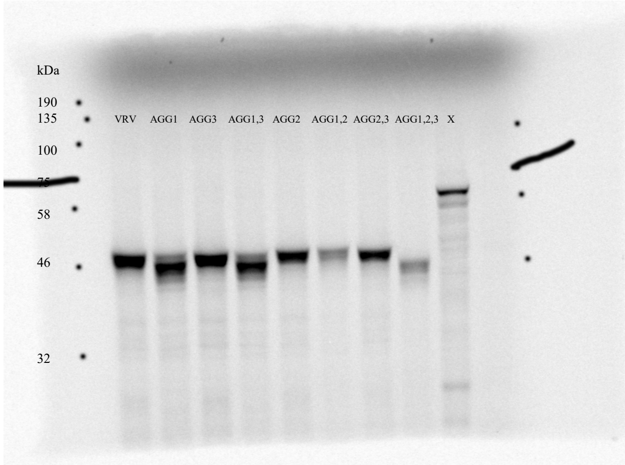

Translation reactions were run on SDS-PAGE and radioactive translation products were imaged with a Cyclone 5 (Packard instrument company) (3 days exposure). The non-radioactive Mr markers do not appear on the exposure but were manually added by aligning the exposure image with the Coomassie blue stained gel (exposure printed on a transparency sheet and superimposed over the Coomassie blue stained gel for perfect alignment). Molecular mass markers are indicated by the dots (for the 190, 135, 100, 58, 46 and 32 kDa markers) and the line (for the 75 kDa marker that corresponds to the thicker band)

Raw image Fig. 3B
Second panel (transcripts)

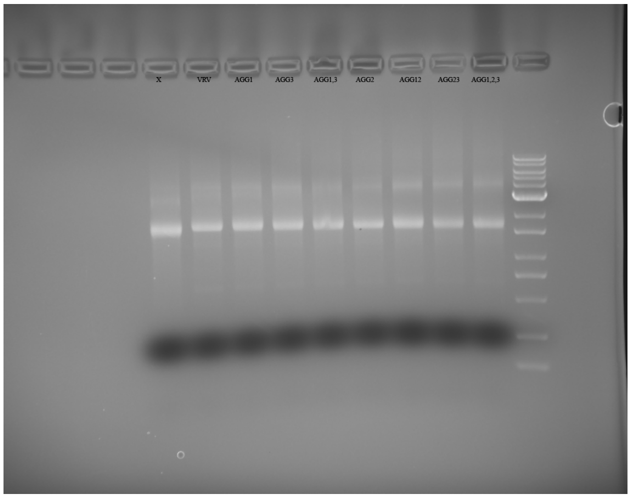

Transcripts were run on a non-denaturing agarose gel to check their integrity (absence of visible degradation). The gel was stained with Ethidium Bromide. The marker on the right lane is the DNA 1kb+ ladder from Froggabio (https://www.froggabio.com/1kb-plus-dna-ladder.html). Because this is a non-denaturing gel, the single-strand RNAs are expected to migrate at the position of a double-strand DNA molecule of approximately half the size, although this can vary from gel to gel depending on the amount of heat generated during the run which can influence the folding status of the single-strand RNA. In this gel, the VRV transcript (~3000 nts) runs approximately at the position of the 1500 bp marker (second band below the 3000 bp thicker band). The nature of the weak upper bands is not clear. They may correspond to different structures (alternate folding) of the RNAs or possibly to small amounts of runaway transcription due to the incomplete restriction enzyme digestion of the constructs.

Raw image Fig. 3B
Third panel (anti-Rluc)

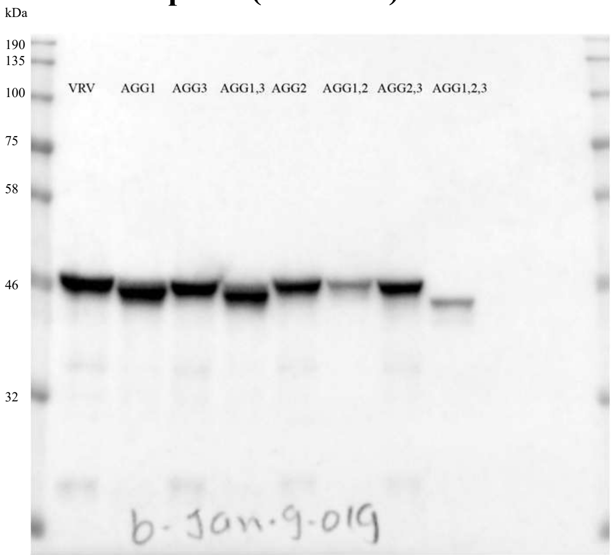

Western blots were imaged as indicated in Material and Methods (15 sec exposure of the blot superimposed with the colorimetric imaging of the Mr marker using the ChemiDoc XRS from BioRad).

Raw image Fig. 3B
Fourth panel (transcripts)

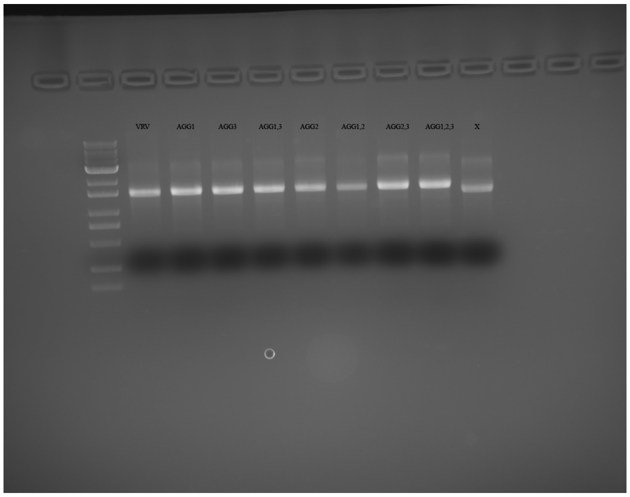

Transcripts were run on a non-denaturing agarose gel to check their integrity (absence of visible degradation). The marker on the left lane is the DNA 1kb+ ladder from Froggabio (https://www.froggabio.com/1kb-plus-dna-ladder.html). The nature of the weak upper bands is not clear. They may correspond to different structures (alternate folding) of the RNAs or possibly to small amounts of runaway transcription due to the incomplete restriction enzyme digestion of the constructs.

Raw image Fig. 4C
Top panel (anti-Rluc)

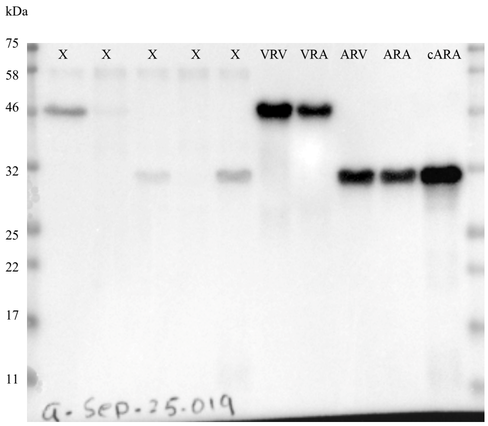

Western blots were imaged as indicated in Material and Methods (1 sec exposure of the blot superimposed with the colorimetric imaging of the Mr marker using the ChemiDoc XRS from BioRad).

Raw image Fig. 4C
Bottom panel (transcripts)

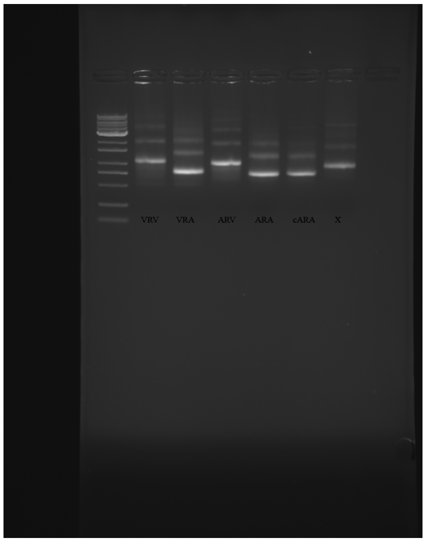

Transcripts were run on a non-denaturing agarose gel to check their integrity (absence of visible degradation). The marker on the left lane is the DNA 1kb+ ladder from Froggabio (https://www.froggabio.com/1kb-plus-dna-ladder.html). Transcript sizes are VRV (~3000 nts), VRA (~1800 nts), ARV (~2800 nts), ARA (~1600 nts) but the single-strand RNA transcripts migrate faster than the corresponding double-strand DNA marker. The nature of the weak upper bands is not clear. They may correspond to different structures (alternate folding) of the RNAs or possibly to small amounts of runaway transcription due to the incomplete restriction enzyme digestion of the constructs.

Raw image Fig. 4F
Top panel (anti-Rluc)

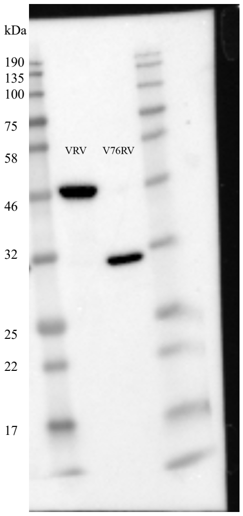

Western blots were imaged as indicated in Material and Methods (1 sec exposure of the blot superimposed with the colorimetric imaging of the Mr marker using the ChemiDoc XRS from BioRad).

Raw image Fig. 4F
Bottom panel (transcripts)

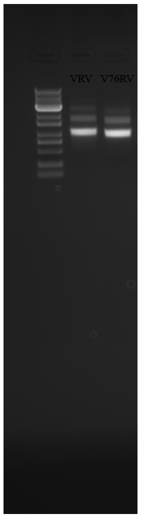

Transcripts were run on a non-denaturing agarose gel to check their integrity (absence of visible degradation). The marker on the left lane is the DNA 1kb+ ladder from Froggabio (https://www.froggabio.com/1kb-plus-dna-ladder.html). Transcripts size are VRV (~3000 nts) and V76RV (~2640 nts). The nature of the weak upper bands is not clear. They may correspond to different structures (alternate folding) of the RNAs or possibly to small amounts of runaway transcription due to the incomplete restriction enzyme digestion of the constructs.
